# Oral artificial microbio-vaccine triggers synergistic immunogenic cell death induction and tumor microenvironment remodeling for liver cancer therapy

**DOI:** 10.1016/j.apsb.2026.06.058

**Published:** 2026-07-04

**Authors:** Qian Cheng, Siyao Li, Lu Deng, Li Xiong, Huiling Hu, Jingjing Li, Lele Zhang, Boda Guo, Chuan Zheng, Jinming Zhang

**Affiliations:** a Chinese Medicine Germplasm Resources Innovation and Effective Uses Key Laboratory of Sichuan Province, Chengdu University of Traditional Chinese Medicine, Chengdu 611130, China; b TCM Regulating Metabolic Diseases Key Laboratory of Sichuan Province, Hospital of Chengdu University of Traditional Chinese Medicine, Chengdu 610072, China; c Department of Urology, Peking University Third Hospital, Beijing 100191, China; d School of Preclinical Medicine, Chengdu University, Chengdu 610106, China; e Department of Rehabilitation Sciences, Faculty of Health and Social Sciences, Hong Kong Polytechnic University Hong Kong 999077, China

**Keywords:** Artificial cells, Oral delivery, Vaccine, Immunogenic cell death, Immunotherapy, Self-assemble, Liver cancer, Tumor microenvironment

## Abstract

Artificial cellular systems, engineered to mimic biological functions, offer a promising platform for advanced drug delivery and immunotherapy. Translating such systems into effective oral tumor vaccines, however, remains challenging due to the harsh gastrointestinal environment, inefficient immune activation, and the toxicity of potent immunogenic agents. To overcome these barriers, we engineered an oral artificial micro bio-vaccine *via* a hierarchical assembly strategy. Triptolide (TP), an immunogenic cell death inducer, was co-assembled with glycyrrhizic acid (GA), an immunomodulator, into a nanodetoxifying core to facilitate efficient loading into hollow *Saccharomyces cerevisiae* microcapsules (YC), forming the final aggregate (TP/GA-NYPs). This rational design leverages the YC for gastric protection and gut mucosa-targeting, while the Chitosan shell ensures robust encapsulation. Crucially, the TP/GA core demonstrates a synergistic interplay, in which TP potently induces immunogenic cell death, while GA concurrently remodels the immunosuppressive tumor microenvironment by suppressing metastasis-related markers and enhancing immunostimulatory cytokine profiles. This spatiotemporally coupled action within a single oral aggregate elicits robust intestinal mucosal and systemic immunity, effectively suppressing liver cancer progression and metastasis while mitigating the systemic toxicity of TP. This work highlights the therapeutic potential of functional aggregates in orchestrating synergistic anti-tumor immunity and offers a versatile platform for oral immunotherapy.

## Graphical abstract

Yeast wall-coated triptolide/glycyrrhizic acid self-assembled oral vaccine targeting gut–liver axis induce potent mucosal and systemic immunity, reprogram immunosuppressive TME, and overcome injectable limitations to suppress liver cancer metastasis and recurrence.

**Figure:**
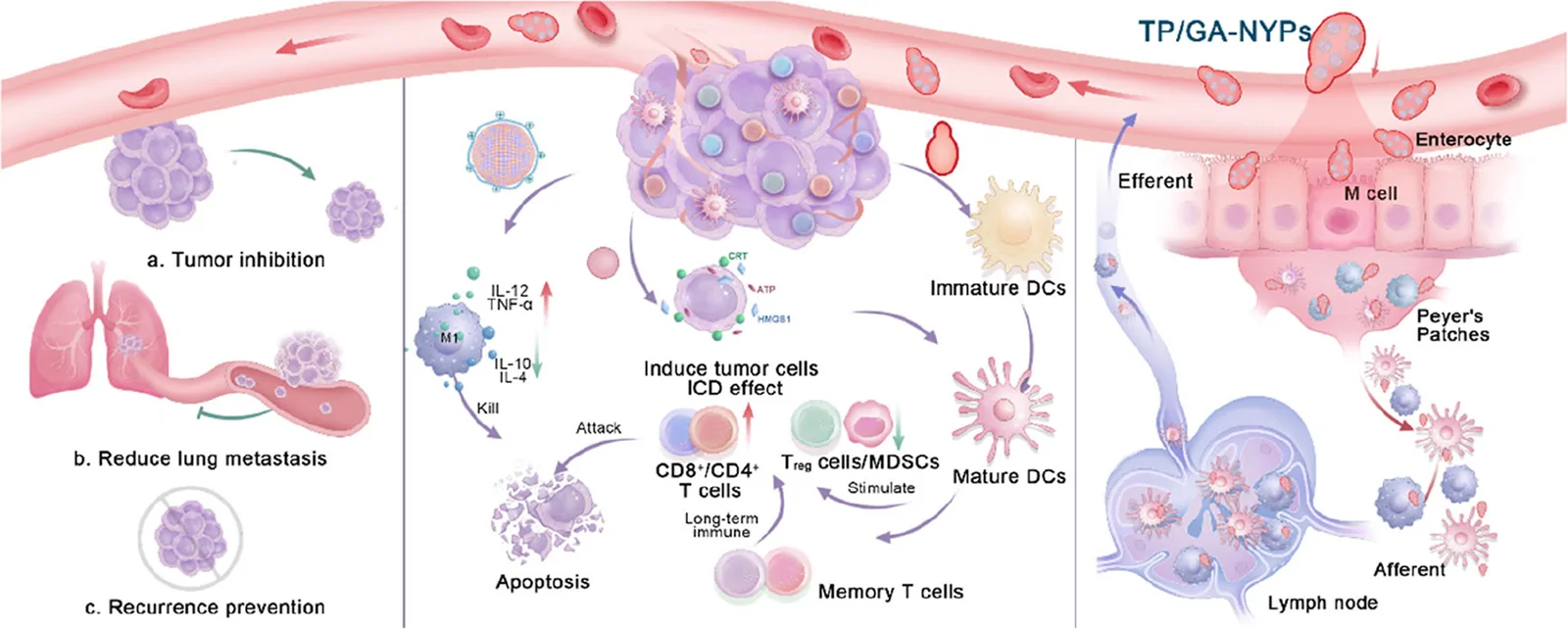

## 1 Introduction

The therapeutic challenges in liver cancer starkly highlight the limitations of current modalities. The immunosuppressive tumor microenvironment (TME) of liver cancer renders it notoriously resistant to treatments^1^. While immune checkpoint inhibitors have marked a paradigm shift, their efficacy is limited by primary resistance in many patients and the inability to establish long-term immunological memory to prevent recurrence^2^, ^3^, ^4^. Therapeutic tumor vaccines eliminate tumors by activating cytotoxic T lymphocytes, generating a more specific and durable anti-tumor immunity^5^. For instance, BioNTech's *mRNA*-based vaccines, which encode tumor-associated antigens and are delivered *via* intravenous injection, have shown promise in early clinical trials by inducing potent T cell responses^6^. Furthermore, Wang et al.^7^ have explored nanovaccines co-delivering peptide antigens and mRNA-encoded antigens, demonstrating enhanced dendritic cells (DCs) targeting and activation in mouse models. However, most existing platforms rely on injectable administration^8^, ^9^, ^10^, ^11^, which often yields suboptimal efficacy due to poor biodistribution and fails to induce robust mucosal immunity—a critical component for preventing metastases^12^.

As a critical component of the mucosal immune system, the gut-associated lymphoid tissue (GALT) harbors abundant immune cells^13^. Notably, the gut–liver axis creates a unique functional link between the GALT and the liver. Immune cells primed and activated in the GALT can traffic directly to the liver *via* the lymphatic system and portal venous circulation, mediating robust local hepatic immunosurveillance. This functional crosstalk renders oral vaccine delivery an exceptionally promising strategy for liver cancer immunotherapy, as it can elicit potent mucosal immune responses that are inaccessible to conventional systemic vaccination *via* intramuscular or subcutaneous routes^14^, ^15^, ^16^. Compared to injectable vaccines, oral formulations offer non-invasiveness and reduced iatrogenic infection risk^17^. Yet, the oral route presents formidable biological barriers: destructive gastric conditions, the intestinal epithelial barrier, and the challenge of achieving targeted accumulation in tumor tissue^18^, ^19^, ^20^. Critically, potent immunogenic agents (such as immunogenic cell death inducers) often induce severe toxicity, while current vaccines predominantly focus on antigen presentation while neglecting TME reprogramming and metastasis suppression^21^, ^22^, ^23^, ^24^. Thus, developing novel oral vaccines to safely and effectively activate mucosal and systemic immune response, and remodel the immunosuppressive TME is imperative for inhibiting tumor metastasis and recurrence^25^^,^^26^.

Here, we have engineered an oral artificial microbio-vaccine formulation (designated NYP) based on natural, food-grade yeast cell wall (YC) microcarriers, to overcome the core bottlenecks of oral liver cancer immunotherapy. Briefly, self-assembled nanocomplexes of the potent immunogenic cell death (ICD) inducer triptolide (TP) and immunomodulatory glycyrrhizic acid (GA) were chitosan-modified to confer positive surface charge, then encapsulated into YC to fabricate NYP. The YC platform endows NYP with robust resistance to gastrointestinal degradation and specific Peyer's patches targeting *via* microfold cells (M cells) recognition for efficient gut-associated lymphoid tissue translocation^27^, ^28^, ^29^, ^30^. Following entry into the systemic circulation, NYP is preferentially internalized by circulating macrophages, which act as “Trojan horses” to hitchhike the formulation to the tumor site *via* their inherent chemotaxis toward the immunosuppressive TME. Once internalized by tumor cells, NYP is rapidly shuttled to the lysosomal compartment, and achieves pH-responsive on-demand drug release, which synergizes with GA-mediated protection to mitigate TP's dose-limiting gastrointestinal toxicity^31^^,^^32^.The co-delivered components elicit a multi-pronged synergistic anti-tumor cascade: TP triggers robust ICD to prime adaptive immunity; GA cooperates with YC *β*-glucan to promote DCs maturation, downregulate metastasis-related MMP9, and collaborate with TP to remodel the immunosuppressive tumor microenvironment and activating both mucosal and systemic anti-tumor immunity (Fig. 1). Collectively, the NYP oral vaccine system achieves potent inhibition of liver tumor growth, lung metastasis, and tumor recurrence *via* establishing durable immunological memory. This work provides a safe, clinically translatable oral therapeutic-prophylactic vaccine modality for liver cancer, addressing critical limitations of current cancer immunotherapies.

**Figure 1:**
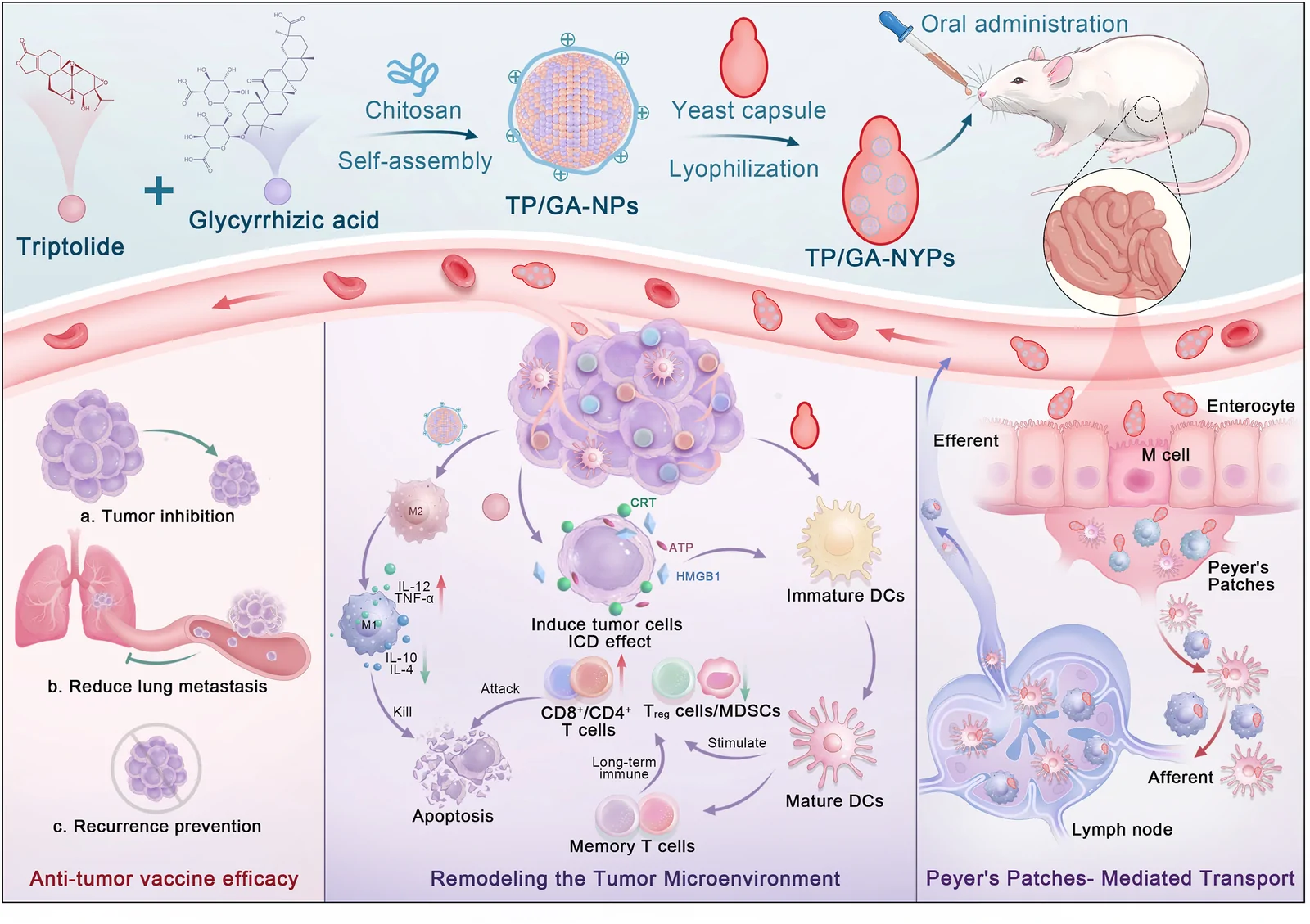
Illustration depicting the process of preparation and mechanism of the oral artificial yeast vaccine (TP/GA-NYPs) for synergistic immunotherapy against liver cancer.

## 2 Materials and methods

### 2.1 Materials

Baker's yeast (*Saccharomyces cerevisiae*) was provided by Lesaffre Management Co., Ltd. (Shanghai, China). TP was obtained from Chengdu Jingcuitiancheng Pharmaceutical Technology (Chengdu, China). GA was obtained from Dalian Meilun Biotechnology (Dalian, China). Chitosan (CS) was provided by Shanghai Macklin Biochemical Technology (Shanghai, China). FITC was purchased from Shanghai Aladdin Biochemical Technology (Shanghai, China). Cyanine5.5 (Cy5.5) was obtained from MedChem Express (Monmouth Junction, NJ, USA). DAPI, annexin V-FITC/PI detection kit, and adenosine triphosphate (ATP) detection kit were purchased from Beyotime Biotechnology (Shanghai, China). Cell Counting Kit-8 (CCK-8) kits were purchased from BioSharp (Hefei, China). Recombinant murine Granulocyte-Macrophage Colony Stimulating Factor (GM-CSF) was purchased from Novoprotein (Suzhou, China). High Mobility Group Box 1 (HMGB1) rabbit monoclonal antibody and Calreticulin (CRT) rabbit monoclonal antibody were obtained from Beyotime (Shanghai, China). FITC-conjugated goat anti-rabbit IgG secondary antibody was purchased from ZENBIO (Chengdu, China). Mouse IL-10, IL-12 p70, IL-4, and TNF-*α* enzyme-linked immunosorbent assay (ELISA) kits were purchased from MultiSciences (Hangzhou, China). Antibodies, including PerCP anti-mouse CD11b, FITC anti-mouse F4/80, PE/Cy7 anti-mouse CD206, FITC anti-mouse CD11c, PE/Cy7 anti-mouse CD80, PE anti-mouse CD86, PE/Cy7 anti-mouse CD3, FITC anti-mouse CD45, PE anti-mouse Ly-6G/Ly-6C (Gr-1), PerCP/Cy5.5 anti-mouse CD4, PE anti-mouse CD25, PE/Cy5 anti-mouse CD8a, PE anti-mouse CD62L, FlTC anti-mouse CD44 were purchased from BioLegend (San Diego, CA, USA).

### 2.2 Cell cultures and animals

The mouse mononuclear macrophage leukemia cells (RAW264.7), mouse hepatocarcinoma cell line (H22), and human hepatocarcinoma cell line (HepG2) were obtained from the Cell Bank of the Shanghai Institute of Biochemistry and Cell Biology, Chinese Academy of Sciences (Shanghai, China). RAW264.7 and HepG2 cells were cultured in DMEM medium (Gibco, Shanghai, China), and H22 cells were cultured in a RPMI 1640 medium (Gibco, Shanghai, China). All of them were supplemented with 10% (*v*/*v*) FBS (Tianhang Biotechnology, Huzhou, China) and 1% (*v*/*v*) penicillin/streptomycin (Boster, Wuhan, China). All cells were cultivated at 37 °C within a 5% CO_2_ water-jacketed incubator (Thermo Fisher Scientific, Waltham, MA, USA).

Immunocompetent male BALB/c mice weighing about 18–20 g were purchased from SPF (Beijing) Biotechnology Co., Ltd. (Beijing, China). All animal experiments involved in this study complied with the ARRIVE guidelines. The animal experiments were conducted under the permission of the Animal Ethics Committee of Chengdu University of Traditional Chinese Medicine (Chengdu, China) (permission number SYXK2020-124), and all experiments were performed in accordance with relevant regulations.

### 2.3 Preparation of NP

TP/GA-NPs (NP) were fabricated based on the solvent injection method. Briefly, TP (2 mg) and GA (4 mg) were dissolved in acetone (1 mL) together, and CS (1 mg) was dissolved in ultrapure water (10 mL). The organic solvent mixture was added dropwise into ultrapure water with CS at 45 °C with a constant rate of 1 mL/min.

### 2.4 Preparation of YC

YC was prepared with baker's yeast according to the previously reported method^33^, ^34^, ^35^. Briefly, 20 g of baker's yeast was dispersed in 200 mL of NaOH (1 mol/L), and the suspension was continuously stirred at 80 °C for 1 h. The insoluble material containing the YC was collected by centrifugation at 424×*g* for 10 min (TGL-25M, BIORIDGE CENTRIFUGE, Shanghai, China). Later on, the sediment was resuspended in 40 mL of pure water, which was adjusted to pH 4–5 with HCl, and incubated for 1 h at 55 °C. Subsequently, the insoluble residue was again collected by the same centrifugal conditions and then washed with 200 mL of pure water once, four times with 40 mL of isopropanol, and twice with 40 mL of acetone. The extracted material was frozen in a −80 °C freezer and then lyophilized to obtain a grey white fine powder.

### 2.5 Fabrication of NYP

TP/GA-NYPs (NYP) were prepared by electrostatic force-driven self-deposition^36^^,^^37^. First, 100 mg YC was incubated with 1 ml of pure water at 37 °C for 30 min. Then, 10 mL NP was added to the solution containing YC. After incubation at 37 °C for 1 h, it was centrifuged at 1696×*g* for 10 min and resuspended it using deionized water. Subsequently, to better encapsulate the drug, the suspension underwent a lyophilization–hydration cycle, repeated twice. Before the last lyophilization, thoroughly wash with deionized water to remove unpackaged NP.

### 2.6 Characterization of NP

High-performance liquid chromatography (HPLC, LC-2030C-3D, Shimadzu, Kyoto, Japan) analysis was employed to measure the drug encapsulation efficiency (EE) and loading efficiency (LE) of TP and GA within NP.

Before HPLC analysis, three times the volume of methanol was incorporated into the NP solution, followed by sonication for 15 min to disrupt the nanostructure and release drugs thoroughly. The mobile phases for HPLC analysis to determine TP and GA were acetonitrile/water (*v*/*v*, 46:54%) and methanol/0.1% phosphoric acid aqueous solution (*v*/*v*, 75:25%), respectively^38^. The EE and LE of the drugs were calculated according to Eqs. (1), (2):

(1) EE (%) = (Amount of drug in nanoparticles)/(Amount of TP/GA added) × 100

(2) LE (%) = (Amount of drug in nanoparticles)/(Total amount of drug added) × 100

The average hydrodynamic particle size (nm), polydispersity index (PDI), and zeta potential (mV) of NP were measured using dynamic light scattering with a Particle Analyzer Lite sizer 1090 (Anton Paar, Graz, Austria). The calculation of the average and standard deviations (SDs) of the diameter or PDI was repeated three times.

The morphology of NP was precisely viewed by transmission electron microscopy (TEM, JEM 1200X, JEOL, Tokyo, Japan). A diluted NP suspension drop was delicately placed on a carbon-coated copper grid and then stained with uranyl acetate and analyzed *via* TEM.

### 2.7 Characterization of NYP

The particle sizes of YC and NYP were determined through a micron particle size analyzer (Bettersizer 2600, Bettersize Instruments, Dandong, China). The zeta potential measurement was carried out in the same way as that for NP, using the Particle Analyzer Lite sizer 1090.

Untreated yeast cells were dispersed in a minimal volume of water first and subsequently subjected to freeze–drying in conjunction with YC and NYP. Subsequently, a scanning electron microscope (SEM, ZEISS Gemini 300, ZEISS, Oberkochen, Germany) was employed to characterize the morphological features of the resultant samples meticulously. To optimize imaging quality, the samples were sputter-coated with gold under vacuum conditions prior to imaging acquisition.

For the purpose of scrutinizing the encapsulation status of NP within YC, the aforementioned three sets of samples were further analyzed and characterized using TEM.

For the determination of EE and DL, the sample treatment and detection methods were identical to those described in 2.6. section, and the following Eqs. (3), (4) were used for calculation:

(3) EE (%) = (Amount of drug in NYP)/(Amount of drug added) × 100%

(4) LE (%) = (Amount of drug in NYP)/(Total amount of drug and YC added) × 100%

The fluorescent dye calcofluor white can bind to glucan in the YC. To visually confirm whether NP are loaded into the empty YC or not, we labeled the NP with FITC and then loaded them into the YC labeled with calcofluor white. Subsequently, confocal laser scanning microscopy (CLSM, TCS SP8 SR, Leica, Wetzlar, Germany) was utilized to observe the co-localization of NP and YC.

Then we evaluated the stability of NYP in simulated gastric fluid (SGF) and simulated intestinal fluid (SIF) by referring to the methods reported previously^29^^,^^39^. The NYP loaded with TP was mixed with SGF (pH 2) or SIF, and then incubated in a 37 °C shaker (100 rpm) for 4 h. After freeze–drying, the morphology of NYP was observed using a scanning electron microscope. Furthermore, NP and NYP suspensions (1 mL) containing the equivalent amount of TP were placed in a dialysis bag (MWCO 3500 Da) against different buffers (15 mL, SIF, SGF) containing 1% Tween 80. They were incubated in a shaking bath (100 rpm) at 37 °C. At various time intervals, release medium (0.5 mL) was collected, and fresh medium in an equal volume was added. With the help of the aforementioned HPLC analysis technique, the TP concentration in the release medium was identified, and the cumulative drug release was determined. The release profiles of TP and GA were similarly evaluated using the same method in conventional buffer media at pH 1.2, pH 4.5, and pH 7.4, respectively.

### 2.8 Toxic effects of drugs on cells

The CCK-8 kit was used to evaluate the cytotoxicity of TP, NP, and NYP to H22 cells. Briefly, H22 was seeded in the 96-well plates (with 1×10^4^ cells per well) and incubated in DMEM or RPMI 1640 medium for a certain period. The cells were cultured for 24 h with TP, NP, and NYP samples prepared in low serum medium (0.5% FBS) with equal amounts of TP (0–1000 nmol/L). Subsequently, we added the CCK-8 detection reagent as required by the instructions. The absorbance at 450 nm was measured and documented through a microplate reader (Molecular Devices, San Jose, CA, USA). Using the same method, the cytotoxicity of TP and NYP was assessed in Caco-2 and RAW264.7 cells.

### 2.9 Cellular uptake in cells

To evaluate the cellular uptake efficiency of NYP, the uptake of NYP by HepG2 was assessed. We added FITC additionally during the preparation process of NP to label the NP. Subsequently, we loaded it into NYP to obtain FITC-NYP. HepG2 cells (1×10^5^ cells per well) were seeded into 12-well plates and confocal dishes. After the cells adhered to the plates or dishes, the culture medium was replaced with the medium containing FITC-NP and FITC-NYP with the same FITC concentration. The cells were co-incubated for different durations. After being collected by trypsin digestion, the cells were resuspended in PBS, cellular uptake was visualized using CLSM, and the fluorescence intensity was quantified by flow cytometry (FCM, FACS CantoII, Becton Dickinson, Franklin Lakes, NJ, USA). Using the same method, the uptake efficiency was further investigated in RAW264.7 cells.

### 2.10 Apoptosis analysis *via* flow cytometry

Cell apoptosis induced was detected by an Annexin V-FITC/PI detection kit^40^. H22 cells (1×10^5^ cells per well) were seeded in twelve-well plates and then treated with GA, TP, NP, YC, and NYP at equivalent concentrations of 50 nmol/L TP for 24 h. The culture medium without adding drugs was used as a control. After incubation for 24 h, the cells were collected and stained with Annexin V-FITC and PI according to the manufacturer's protocol. Finally, all samples were analyzed using FCM.

### 2.11 ICD effect of NYP *in vitro*

To evaluate the ICD effect, H22 tumor cells (2 × 10^5^ per well) were seeded into 12-well plates. After treatments with the various materials (Saline, GA, TP, NP, YC, NYP) for 24 h, the cells were subjected to a routine immunofluorescence examination to assess CRT surface exposure and HMGB1 release. Upon collection, the cells were washed with PBS and fixed with 4% PFA. Subsequently, we fixed the cells on adhesive glass slides (Beyotime, Shanghai, China), two slides per group. The two parts of the cells were respectively stained with HMGB1 rabbit monoclonal antibody (1:100) and CRT rabbit monoclonal antibody (1:100) and then incubated overnight at 4 °C. It should be emphasized that the cells intended for incubation with the HMGB1 antibody were initially subjected to permeabilization using 0.1% Triton at room temperature for 15 min before the incubation process. The next day, they were stained with FITC-conjugated goat anti-rabbit IgG secondary antibody (1:1000). Then, the samples were mounted with an anti-fluorescence quenching agent containing DAPI. Fluorescence images were acquired *via* CLSM. Meanwhile, the ATP kit was used to measure the amount of ATP inside the cells. According to the manufacturer's instructions, we detected the intracellular ATP content by chemiluminescence method at a wavelength of 560 nm on a multi-functional microplate reader (Molecular Devices, San Jose, CA, USA). Using the same method, the ICD-inducing capability of NYP was compared with that of DOX in H22 cells, and HMGB1 release, CRT exposure, and ATP release were further examined in HepG2 cells.

### 2.12 Activation of BMDCs

Bone marrow-derived DCs (BMDCs) were generated from bone marrow cavities of 7–8-week-old C57BL/6 mice reported previously. After the animals were euthanized, they were put in 75% ethanol for disinfection for 30 min. Then the bones were collected and placed into 75% ethanol for 2 min, and afterward washed in PBS. Both distal ends of the bones were cut off, and the bone marrow was collected by flushing with RPMI 1640 medium. Red blood cells were lysed by incubating the bone marrow preparation in ACK lysis buffer for 2 min. The remaining cells were collected by centrifugation at 300×*g* for 5 min, and then grown in 12-well plates (1 × 10^6^ cells per well) in RPMI 1640 medium supplemented with 10% fetal bovine serum, 1% penicillin/streptomycin, 10 ng/mL IL-4, and 20 ng/mL GM-CSF. Fresh medium was added on Day 3. On Day 6, the nonadherent cells were aspirated and incubated in 12-well plates with fresh medium and were treated with RPMI 1640 medium, GA, TP, NP, YC, and NYP. Following 24 h of incubation, BMDCs were collected and stained with FITC anti-mouse CD11c, PE/Cy7 anti-mouse CD80, and PE anti-mouse CD86, measured by FCM.

### 2.13 Biodistribution studies

To establish an H22 tumor-bearing BALB/c mouse model for evaluating the tumor-targeting capability of NYP, 3 × 10^6^ H22 cells were subcutaneously inoculated into male BALB/c mice. Following successful tumor establishment, the mice were randomly allocated into three groups (*n* = 3) and administered orally with free Cy5.5, Cy5.5-NP, or Cy5.5-NYP (0.6 mg/kg), respectively. *In vivo* imaging was performed at predetermined intervals (0.5, 1, 2, 4, 10, and 24 h) using an IVIS Lumina imaging system (PerkinElmer, Waltham, MA, USA). After 24 h, the mice were euthanized, and major organs (heart, liver, spleen, lungs, and kidneys), Peyer's patches, mesenteric lymph nodes (MLN), and tumors were harvested for *ex vivo* fluorescence imaging. Quantitative analysis of fluorescence intensity was conducted using Living Image Software Version 4.5. Additionally, frozen sections of tumor tissues and Peyer's patches were prepared to visualize drug distribution through fluorescence microscopy.

To further investigate the biodistribution of NYP after oral administration, H22 tumor-bearing mice were orally administered with NP and NYP separately. The mice were euthanized at 0, 2, 4, 12, and 24 h post-administration, followed by collection of the ileum, colon, and tumor tissues. The harvested tissues were snap-frozen in liquid nitrogen and prepared as frozen sections. The spatial distribution of TP in these tissues was subsequently analyzed using mass spectrometry imaging.

### 2.14 Immune activation and anti-tumor effects *in vivo*

A tumor xenograft model was established with H22 cells. H22 cells (3 × 10^6^/100 μL, respectively) were inoculated in the lower right side of the back of BALB/c mice. Six days later, mice were randomized to six groups (*n* = 9) and received different treatments. The drugs were administered orally by gavage. Saline, TP, GA, NP, YC, and NYP (TP dose of 0.3 mg/kg) were administered four times, on Days 0, 4, 8, and 12. The first administration day was defined as Day 0. Mouse survival, tumor growth, and body weight were continuously monitored. The tumor volume was calculated according to Eq. (5):

(5) Tumor volume = Length × 1/2 Width^2^

Tumor volume thresholds are set at 2000 mm^3^ as the mandatory sacrifice indicator. Of note, for the survival analysis, all mice (*n* = 9 per group) were included from the beginning of the experiment until their respective endpoints. For the terminal measurements (*e.g*., tumor weight and volume), only mice that survived until the scheduled sacrifice at the end of the study were analyzed.

Seven days after the last administration, all mice were sacrificed. The number of Peyer's patches in the small intestine was quantified. The tumors were dissected, weighed, and photographed. Each tumor specimen was divided into multiple segments for distinct analytical purposes. A part of the tumor tissues was lysed, and the supernatant was collected for quantification of IL-10, IL-4, IL-12 p70, and TNF-*α* cytokine levels using commercially available ELISA kits. Some of the tumor tissues were fixed with 4% (*v*/*v*) formalin neutral buffer solution to preserve them, and then the tumor tissue slices were acquired using cryoembedding. Subsequently, H&E, TUNEL, MMP9, VEGF, and CD31 staining procedures were performed to further evaluate the tumor inhibition, apoptosis, and angiogenesis suppression efficacy. To further evaluate the inhibitory potential against tumor metastasis, immunofluorescence staining of EMT-related markers (N-cadherin and E-cadherin) was performed. Additionally, double staining of cGAS and STING was carried out to investigate the molecular mechanisms of TP-induced ICD.

Lung specimens from each experimental group were collected, intratracheally perfused with India ink (Leagene Biotechnology, Beijing, China; 1:5 PBS dilution), and subsequently fixed in 10% neutral buffered formalin. Photographic documentation of ink-stained pulmonary foci was performed and quantified by direct manual counting.

### 2.15 ICD and immune activation effect *in vivo*

To validate the impact of ICD induction *in vivo*, tumor tissues were used to analyze CRT and HMGB1 expression *in vivo via* immunofluorescence.

Tumor tissues were enzymatically digested into single-cell suspensions, followed by flow cytometric analysis of immune cell infiltration using fluorochrome-conjugated antibodies specific for DCs (CD11c^+^CD80^+^CD86^+^), CD4^+^ T cells(CD45^+^CD3^+^CD4^+^), CD8^+^ T cells (CD45^+^CD3^+^CD8^+^), myeloid-derived suppressor cells (MDSCs) (CD45^+^CD11b^+^Gr-1^+^), tumor-associated macrophages (TAMs) (CD11b^+^F4/80^+^CD86^+^/CD206^+^), and regulatory T cells (Tregs) (CD45^+^CD3^+^CD4^+^CD24^+^). Single-cell suspensions derived from spleen and tumor draining lymph nodes (TDLNs) were also immunophenotyped by multicolor FCM to quantify DCs (CD11c^+^CD80^+^CD86^+^), CD4^+^ (CD45^+^CD3^+^CD4^+^), and CD8^+^ (CD45^+^CD3^+^CD8^+^) T cell populations.

### 2.16 Immunization and antitumor efficacy in the anti-recurrence model

The same modeling method was built as described previously. H22 cells (3 × 10^6^/100 μL, respectively) were inoculated in the lower right side of the back of BALB/c mice. Six days later, the mice were randomized into six groups and received different treatments. The drugs were administered orally by gavage. Saline, TP, GA, NP, YC, and NYP (TP dose of 0.3 mg/kg) were administered four times, on Days 0, 3, 6, and 9. The first administration day was defined as Day 0. On Day 11th, tumor resection surgery was performed on tumor-bearing mice, leaving approximately 10% of the residual tumor tissue to verify the ability of NYP to inhibit tumor recurrence.

Mouse survival, tumor growth, and body weight were recorded every other day. Twelve days after the resection surgery, all mice were sacrificed (*n* = 5). Tumor cells, lymphocytes in tumor-draining lymph nodes, and splenocytes were collected on Day 24 and stained with anti-mouse CD3, CD4, CD8, CD44, CD62L, CD86, CD80, and CD11c antibodies. The effector memory T cells (CD3^+^CD4^+^CD44^+^CD62L^–^, CD3^+^CD8^+^CD44^+^CD62L^–^) and mature DC population (CD11c^+^CD80^+^CD86^+^) were analyzed by FCM.

### 2.17 Safety evaluation of the oral vaccines

The biosafety studies were carried out on healthy BALB/c mice. mice were randomly divided into six groups (*n* = 5) and injected intravenously with Saline, TP, GA, NP, YC, and NYP (TP dose of 0.3 mg/kg) every 2 days for 4 times, respectively. Then, the body weights of the mice were monitored every 2 days.

On the 13th day, whole blood samples were collected and analyzed for white blood cells (WBCs), platelets (PLTs), lymphocytes, monocytes, hemoglobin (HGB), granulocytes, and red blood cells (RBCs) concentration using automated hematology analyzers (XN-1000, Sysmex, Kobe, Japan). Serum samples were collected for the hematological assay to detect the levels of blood urea nitrogen (BUN), creatinine (CRE), aspartate aminotransferase (AST), and alanine aminotransferase (ALT). Besides, the major organs, including the heart, liver, spleen, lung, and kidneys, were collected for H&E staining. Additionally, gastric, ileal, and colonic tissues were collected and processed into H&E staining sections to evaluate the protective effects of orally administered NYP on the gastrointestinal tract of mice.

### 2.18 Statistical analysis

The data are presented as mean ± standard deviation (SD). Normality and homogeneity of variances were tested for all datasets. A one-way two-sided analysis of variance (ANOVA) was applied assuming normality and homogeneity of variances. If these assumptions were violated, the Kruskal–Wallis H test was used instead. When the Kruskal–Wallis test indicated a significant difference, Dunn's *post hoc* test with Bonferroni correction was applied for pairwise comparisons. The statistical significance of the differences among the two groups was assessed by analysis of variance followed by a test based on a value of *P* < 0.05 using GraphPad Prism software Vision 8). Statistical significance was set as follows: ∗*P* < 0.05, ∗∗*P* < 0.01, ∗∗∗*P* < 0.001, and ns denotes no significant difference.

## 3 Results

### 3.1 Preparation and characterization of NYP

We first fabricated TP&GA self-assemble nanoparticles (NP) using the solvent injection method. AutoDock was employed to reveal the mechanism of this assembly through performing molecular dynamics simulations on the binding capability between TP and GA. The results were typically evaluated based on binding energy to determine docking success. As shown in Fig. 2A, the two molecules formed four hydrogen bonds at the docking site, with an optimal binding energy of −4.0 kcal/mol, indicating a stable interaction between TP and GA. NP exhibited a monodisperse size profile with a mean hydrodynamic diameter of 139.25 ± 0.63 nm (PDI <0.2), as determined by dynamic light scattering (Fig. 2B). TEM image (Fig. 2B, top left corner) indicated that the NP displayed a spherical or quasi-spherical morphology. To achieve effective encapsulation within YC, surface modification of nanoparticles with chitosan resulted in a complete reversal of Zeta potential from −24.44 ± 1.19 mV to 33.76 ± 1.52 mV (Fig. 2C), achieving cationic surface charge conversion and facilitating electrostatic interaction-driven self-assembly with negatively charged YC. The encapsulation efficiency of TP and GA in NP are 99.65 ± 2.25% and 91.63 ± 4.59%, respectively, and the loading capacity of TP and GA in NP are 28.95 ± 0.19% and 53.47 ± 1.80%, respectively. Fig. 2D illustrates the fabrication process of nanoparticle-loaded yeast capsules (NYP), where yeast capsules were loaded with NP *via* synergistic integration of electrostatic self-assembly and aqueous hydration–lyophilization cycles. As the result showed in Fig. 2E, YC exhibited a negative surface charge with a zeta potential of −9.81 ± 0.32 mV. Upon loading with cation-modified nanoparticles, the zeta potential reversed to a positive value of 8.81 ± 1.63 mV, indicating the drug loaded nanoparticles were successful loaded into yeast capsules, while the particle size after nanoparticles loading remained basically unchanged (Fig. 2F–H). SEM and TEM micrographs demonstrated distinct morphological features (Fig. 2I): yeast cells exhibited smooth spherical surfaces, whereas yeast capsules displayed irregular porous architectures indicative of cytoplasmic component removal. NYP presented a relatively plump morphology, further demonstrating successful encapsulation of NP within the YC. To visually confirm the encapsulation of NP within YC, fluorescent staining with Calcofluor White, a *β*-glucan-specific dye targeting the YC, was employed for selective CLSM imaging. As shown in Fig. 2J–a characteristic core–shell architecture was observed, wherein FITC-labeled NP exhibited homogeneous distribution within the YC core, providing direct evidence of successful NP loading. After encapsulation into YC to form NYP, the encapsulation efficiency of TP was 65.26 ± 4.60%, while the drug loading capacity was 1.78 ± 7.60%, and the encapsulation efficiency of GA was 62.31 ± 5.18%, and the drug loading capacity was 2.71 ± 7.47% (Supporting Information Table S1). The pivotal challenge in oral drug delivery systems lies in ensuring that nanoparticles can maintain structural integrity while traversing the harsh gastric environment. To investigate whether YC could protect NP from the harsh gastrointestinal environment, SIF and SGF were employed to assess the stability of NYP following oral gavage. As anticipated, NYP maintained structural integrity after 4 h incubation in SGF (pH 1.2) and SIF (pH 6.8), confirmed by SEM (Fig. 2K). As shown in Supporting Information Fig. S1, the cumulative release of both TP and GA from NYP in SGF and SIF remained relatively low over 24 h, when compared with NP. These findings indicated that NYP remained stable during gastrointestinal transit, which was a critical feature for subsequent M cell-mediated transport. Furthermore, the release profiles of NYP in standard buffer solutions at pH 1.2, 4.5, and 7.4, are provided in Supporting Information Fig. S2. The results demonstrated that NYP exhibited minimal cumulative release of TP and GA at pH 1.2 and pH 7.4 over 24 h, indicating good stability in gastric and circulatory environments. At pH 4.5, NYP showed sustained and significantly higher release, with approximately 67.84% of TP and 71.14% of GA released within 24 h. Notably, compared with uncoated NP, NYP displayed a slower release profile at pH 1.2, confirming the effective protective role of YC shell in preventing premature drug leakage in the acidic gastric environment. This release behavior is consistent with the intended design of NYP, where drug release is preferentially triggered within acidic intracellular compartments (*e.g.*, phagolysosomes) after tumor cellular uptake, rather than in the gastrointestinal tract or bloodstream.

**Figure 2:**
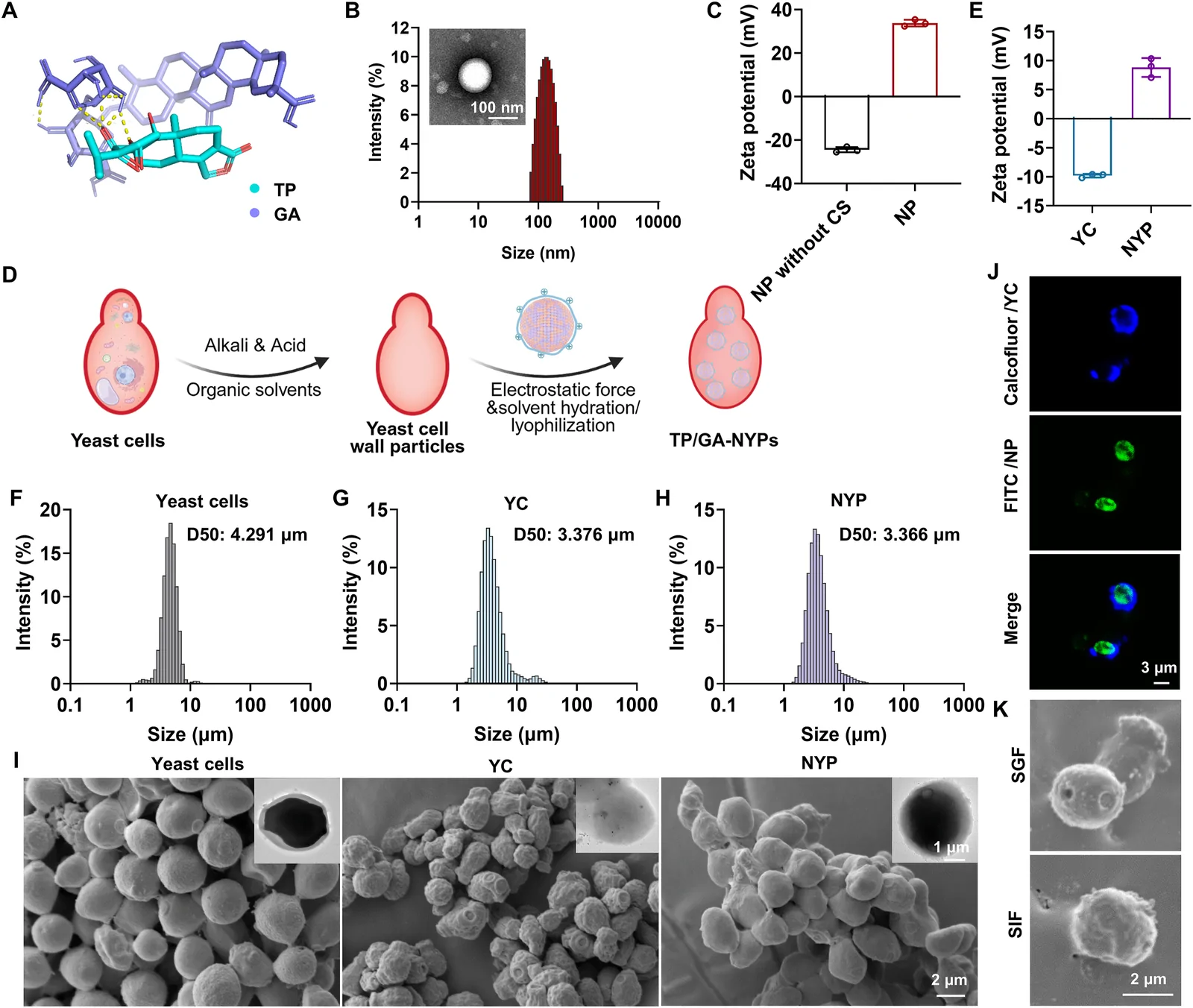
Preparation and characterization of NYP. (A) Molecular interaction of the NP. (B) The size distribution and TEM images of NP. (C) Zeta potential of TP/GA-NPs and nanoparticles without CS. (D) Preparation diagram of NYP. (E) Zeta potential of YC and NYP. (F–H) size distribution of yeast cells, YC, and NYP. (I) SEM and TEM images of yeast cells, YC, and NYP. (J) Representative CLSM images of FITC-NPs (green), and YC was labeled with calcofluor white (blue). (K) SEM images of NYP after SGF and SIG treatment. Data are presented as mean ± SD. Statistical significance was determined by A one-way two-sided ANOVA, *n* = 3.

### 3.2 *In vitro* immune activation analysis of NYP

In this study, TP was chosen as a model chemotherapeutic agent to induce ICD, which can kill tumor cells and facilitate the uptake, processing, and presentation of tumor antigens by APCs to improve T cell responses. First, we evaluated cellular uptake of FITC-labeled NYP in tumor cells and macrophages. CLSM and flow cytometric quantification (Supporting Information Figs. S3 and S4) revealed efficient uptake by both cell types after 4 h, with 80.13% of RAW264.7 and 69.23% of HepG2 cells exhibiting positive fluorescence. Then CCK-8 assay was conducted to evaluate the cytotoxicity of NYP against tumor cells, intestinal epithelial cells and immune cells *in vitro*. As shown in Fig. 3A, TP, NP, and NYP all decreased the cell proliferation of H22 cells; the IC_50_ values were determined as 44.21 ± 0.96 nmol/L (TP), 40.27 ± 0.98 nmol/L (NP), and 41.73 ± 0.98 nmol/L (NYP), respectively, after incubation for 24 h. The slightly attenuated cytotoxicity of NYP may be attributed to the encapsulating effect of YC on NP. For the potential toxicity of orally delivered TP to intestinal epithelial cells and immune cells in Peyer's patches, we evaluated the cytotoxic effects of free TP and NYP using Caco-2 and RAW264.7 as representative cell models. The CCK-8 assay results, presented in Supporting Information Fig. S5, demonstrated that high-concentration of free TP exhibited pronounced cytotoxicity at the tested concentrations, but low dose of NYP significantly reduced its toxicity to both cell types. Subsequently, the *in vitro* apoptosis effect was further assessed by the Annexin-V FITC/PI apoptosis detection kit and a statistical analysis was performed on the total apoptosis rate of each group (Fig. 3B and C). TP, NP and NYP demonstrated significantly elevated apoptosis rates, consistent with cytotoxicity assays, while GA and YC showed minimal pro-apoptotic effects. At the same time, quantitative analysis of total apoptosis revealed 1.4- and 1.2-fold higher rates in NP and NYP groups *versus* free TP. Notably, NYP displayed slightly lower apoptosis than NP, likely attributable to delayed TP release kinetics mediated by the YC coating.

**Figure 3:**
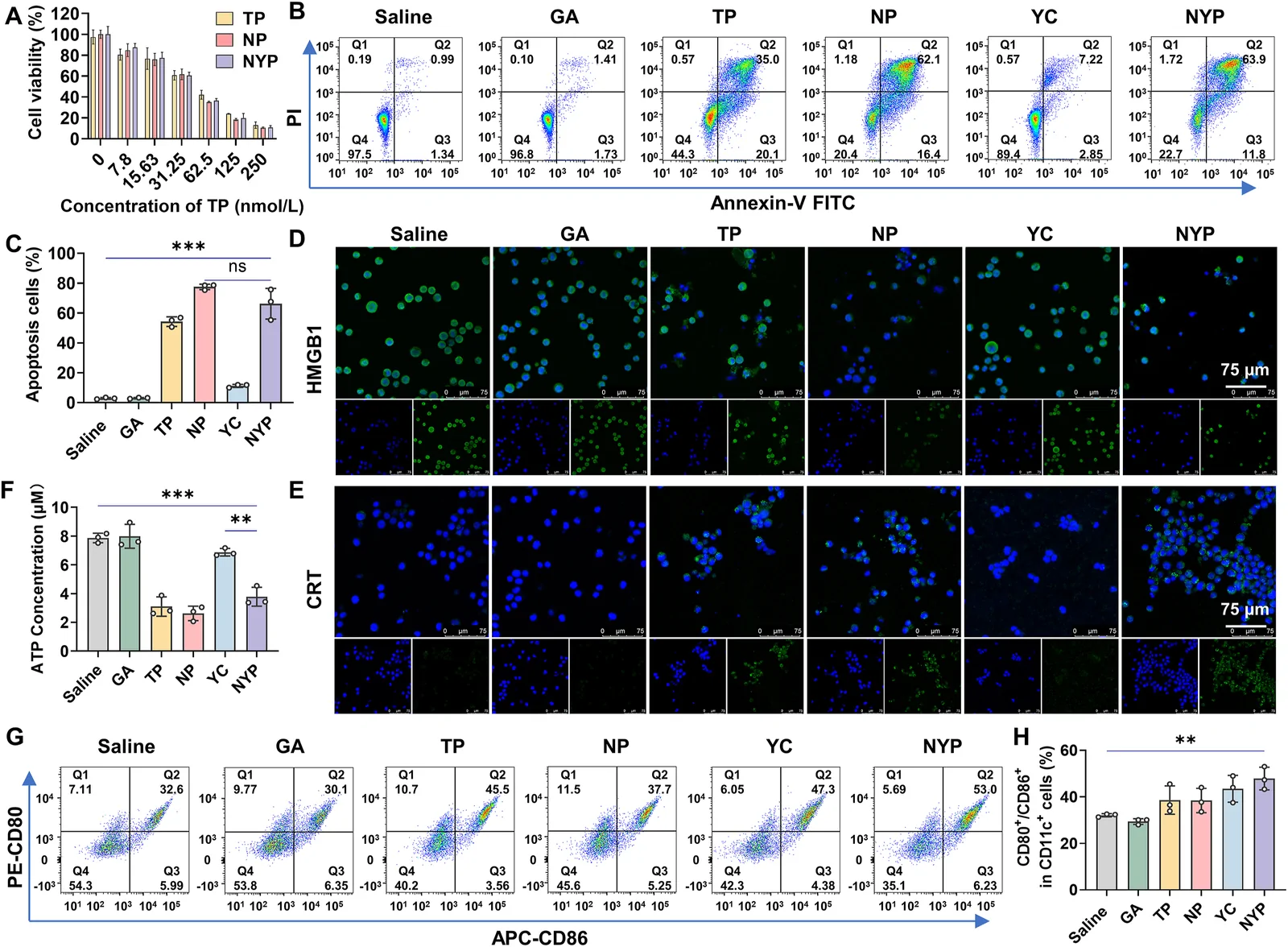
Immune activation analysis *in vitro*. (A) Cytotoxicity against H22 cells after treatment with TP, NP, and NYP after 24 h (*n* = 6). (B) FCM detection of apoptosis following different treatment groups with Annexin V-FITC and PI staining. (C) Quantitative statistical results of apoptotic cell ratios. (*n* = 3). (D) CLSM images of HMGB1 in H22 cells after incubation with different formulations for 24 h. (E) CLSM images of CRT in H22 cells after incubation with different formulations for 24 h. (F) Intracellular ATP concentrations of H22 cells after incubation with different formulations for 24 h, measured by a luminescence-based assay (*n* = 3). Representative FCM images (G) and proportions (H) of CD80^+^ CD86^+^ mature DCs gated on CD11c^+^ DCs after treatment with different formulations for 24 h, respectively (*n* = 3). Data are presented as mean ± SD. Statistical significance was determined by A one-way two-sided ANOVA. Specific *P* values are marked in the figure, with significance levels defined as ∗*P* < 0.05, ∗∗*P* < 0.01, ∗∗∗*P* < 0.001, and ns denotes no significant difference.

Recent studies have demonstrated that ICD triggers the release of damage-associated molecular patterns (DAMPs), including extracellular ATP, surface-exposed CRT, and secreted HMGB1 from dying tumor cells. The release of DAMPs by tumor cells activates DCs, thereby triggering T cell-mediated immune responses^41^^,^^42^. To validate the ICD effects of NYP, the release of HMGB1 and ATP, and the expression of CRT were examined in H22 tumor cells post-drug treatment. As depicted in Fig. 3D, relative to the saline group, CLSM images revealed a marked decrease in nuclear co-localization of blue fluorescence in TP, NP, and NYP treatment groups, indicating a notable release of HMGB1 from the nucleus into the extracellular milieu which will facilitate DCs maturation. As shown in Fig. 3E, the expression of CRT on the H22 cell surface was enhanced after TP, NP, and NYP treatment incubation for 24 h when compared to the saline group. In addition, the content of intracellular ATP levels of TP, NP, and NYP groups was significantly decreased compared to the saline group (Fig. 3F), indicating that NYP was able to induce the release of intracellular ATP to the extracellular space. To verify the universality of the ICD for TP in our NYP vaccine, we have further validated TP-induced ICD in another human liver cancer cell line, HepG2. The results consistently demonstrated that NYP effectively induce CRT exposure, HMGB-1 and ATP release in HepG2 cells (Supporting Information Fig. S6). Notably, NYP induced ATP secretion, CRT exposure, and HMGB1 release at levels comparable to those of commonly used ICD inducer DOX^43^ (Supporting Information Fig. S7). DCs play a vital role in generating tumor specific immune responses, thus, we investigate the maturation of BMDCs treated with different formulations *via* FCM for co-stimulatory molecule upregulation (CD80^+^/CD86^+^). Before FCM analysis, BMDCs were co-cultured for 24 h with the supernatant of H22 cells treated with different formulations. As shown in Fig. 3G and H, compared to TP groups, the YC group showed slightly increased maturation of DCs, while NYP group exhibited the highest proportion of CD80^+^CD86^+^ BMDCs, suggesting the DAMPs released by tumor cells treated with NYP could enhance the maturation of DCs *in vitro*, thus potentially boosting the progression of ICD.

### 3.3 Biodistribution analysis of NYP after oral administration *in vivo*

The tumor-targeting capacity of NYP was further investigated in H22 tumor-bearing mice. To label the NYP with fluorescence, Cy5.5 was first encapsulated into the NP using a similar approach to TP. Subsequently, Cy5.5-labeled nanoparticles (Cy5.5-NP) were encapsulated into YC using the same protocol, yielding Cy5.5-labeled nanocomposite yeast particles (Cy5.5-NYP). When the tumor volume reached approximately 1000 mm^3^, the mice were administered free Cy5.5, Cy5.5-NP, and Cy5.5-NYP orally (*n* = 3 in each group). The mice were imaged using the IVIS Spectrum scanner at different time points (Fig. 4A). At 24 h, the mice were euthanized, and the main organs, Peyer's patch, MLN, and the tumors were harvested and visualized *ex vivo* (Fig. 4B). As shown in *In Vivo* Imaging System image and quantitative analysis in Fig. 4C, free Cy5.5 group exhibited transient gastric accumulation followed by rapid systemic clearance, and limited fluorescence could be found in tumor tissue. Meanwhile, the Cy5.5-NP group showed rapid fluorescence accumulation in tumor regions at 0.5 h post-administration, but short-lived tumor localization, indicating poor tumor retention. In contrast, Cy5.5-NYP achieved sustained and selective tumor accumulation, exhibiting intense fluorescence at 0.5 h after administration and maintained until 24 h, approximately 1.53-fold higher fluorescence intensity than Cy5.5-NP at 24 h post-administration, demonstrating superior tumor-targeted delivery and retention capacity of NYP. Furthermore, the fluorescence signal intensity in the *ex vivo* tumor tissue of mice treated with NYP reached up to 2.08- and 1.65-fold that of the free Cy5.5 and Cy5.5-NP treated group (Fig. 4D). Notably, the NYP group demonstrated significantly higher fluorescence intensity in Peyer's patches compared to the other two groups, confirming the feasibility of nanoparticle translocation through these gut-associated lymphoid tissues.

**Figure 4:**
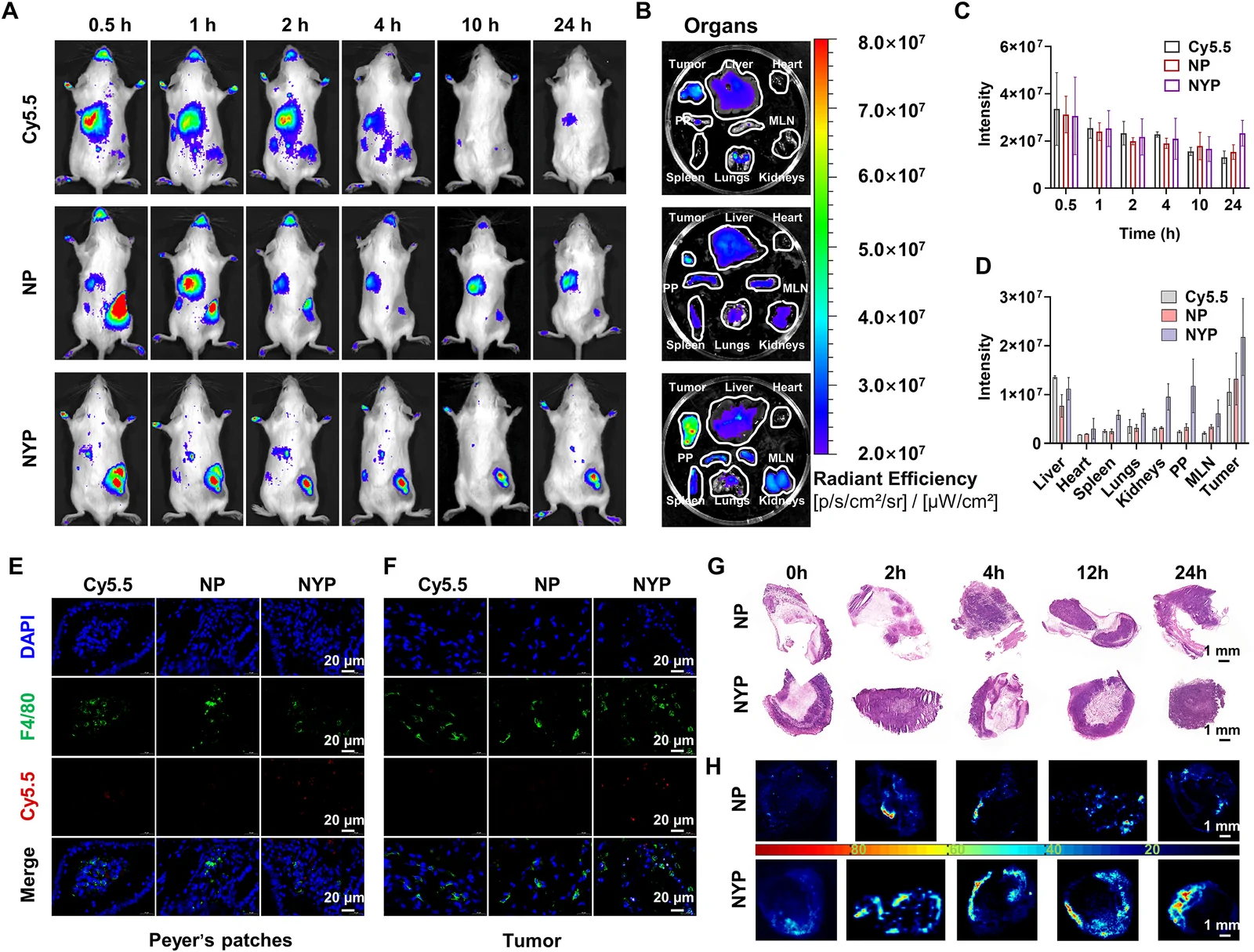
Biodistribution of NYP *in vivo.* (A) *In vivo* real-time imaging of tumor-bearing mice after oral administration of Cy5.5, Cy5.5-NP, and Cy5.5-NYP. (B) *Ex vivo* real-time imaging of tissues after 24 h. (C) Quantitative results of fluorescence intensity of the tumor at different time points after administration (*n* = 3). (D) Quantitative results of fluorescence intensity of isolated tissues (*n* = 3). (E, F) Representative immunofluorescence images of Peyer's patches and tumor at 24 h after oral gavage of free Cy5.5 (green), Cy5.5-NP, and Cy5.5-NYP, respectively. Cell nuclei were stained with DAPI (blue) and macrophages were stained with F4/80 (green). (G, H) MSI images depicting the major fragment ion (*m*/*z* 359.4) of TP are displayed alongside H&E-stained sections at 0, 2, 4, 12, and 24 h following NP and NYP administration, demonstrating TP enrichment within tumor tissue. Data are presented as mean ± SD. Statistical significance was determined by A one-way two-sided ANOVA. Specific *P* values are marked in the figure, with significance levels defined as ∗*P* < 0.05, ∗∗*P* < 0.01, ∗∗∗*P* < 0.001.

Next, fluorescence microscopy analysis of cryosectioned Peyer's patches and tumor tissues revealed distinct Cy5.5 biodistribution patterns of different formulations. As shown in Fig. 4E and F, the NYP group exhibited the strongest fluorescence signal in Peyer's patches, demonstrating efficient translocation *via* gut-associated lymphoid tissues following oral administration. Notably, compared with other groups, the NYP group also showed pronounced Cy5.5 accumulation in tumor sections. This accumulation colocalized with F4/80-labeled macrophages, and the NYP group exhibited the strongest fluorescence signal among all groups. As key phagocytic cells, macrophages can take up drug-loaded particles within Peyer's patches and actively transport them to tumor sites *via* their intrinsic homing capacity toward the tumor microenvironment, thereby functioning as cellular vehicles. The observed co-localization with macrophages provides strong evidence supporting the macrophage-mediated transport and tumor-targeting mechanism of NYP. We further employed mass spectrometry imaging to examine the distribution pattern of TP at the tumor site. As shown in Fig. 4G and H, compared with the NP group, TP in the NYP group exhibited significantly higher accumulation in tumor tissues at all observed time points (2, 4, 12, and 24 h) post-administration. Taken together, these results clearly verified that NYP was capable of active translocation *via* Peyer's patches after oral administration for enhanced tumor retention.

### 3.4 *In vivo* tumor inhibition

Next, we explored whether oral vaccination of NYP could generate antitumor immunity *in vivo*. H22 tumor-bearing BALB/c mice model was established, the mice were randomly divided into six groups (*n* = 9 in each group), and orally administered saline, GA, TP, NP, YC, and NYP, respectively, every four days for a total of four treatments (Fig. 5A).

**Figure 5:**
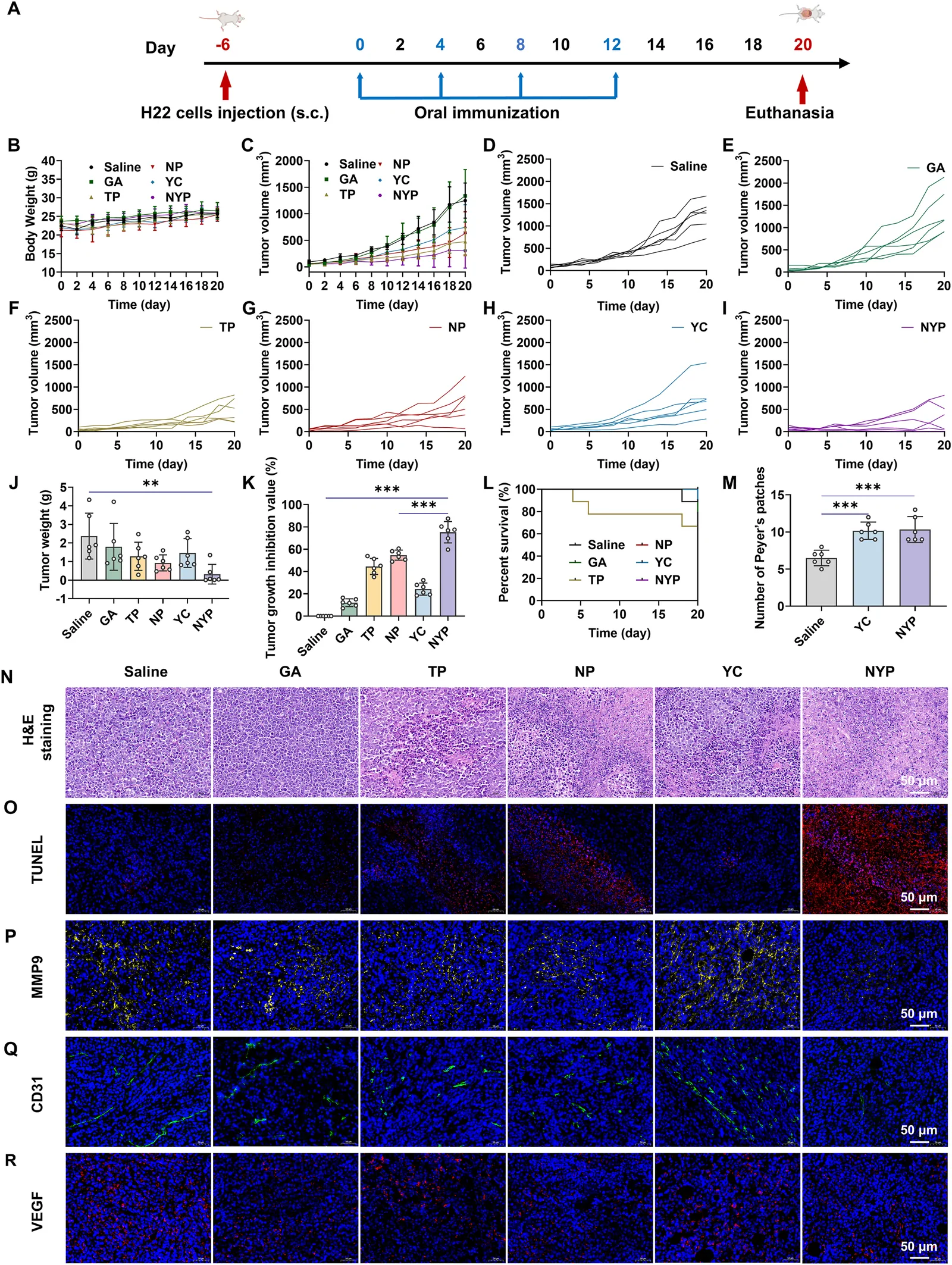
Antitumor efficacy of NYP *in vivo*. (A) Dosing regimens for pharmacodynamic evaluation. (B) Weight growth during the 20-day treatment (*n* = 6). (C) Tumor growth curve (*n* = 6). (D–I) Tumor volume growth curves of mice treated with saline, GA, TP, NP, YC, and NYP groups (*n* = 6). (J) Tumor weight in each group (*n* = 6). (K) Tumor growth inhibition rate (*n* = 6). (L) The survival curves of each group in the H22 tumor model (*n* = 9). (M) Changes in the number of Peyer's patches (*n* = 6). (N–R) H&E staining and immunofluorescence detection of TUNEL (red), MMP9 (yellow), CD31 (green), and VEGF (red) expression in tumor sections, and cell nuclei were stained by DAPI (blue). Data are presented as mean ± SD. Statistical significance was determined by A one-way two-sided ANOVA. Specific *P* values are marked in the figure, with significance levels defined as ∗*P* < 0.05, ∗∗*P* < 0.01, ∗∗∗*P* < 0.001.

The body weight maintained a steady growth in each group, indicating that all formulations were well-tolerated (Fig. 5B). As shown in Fig. 5C–I, compared with the saline group, both the TP and NP groups showed delayed tumor growth. Remarkably, the NYP group significantly slowed down the growth rate of the tumor, with an average tumor volume of 303.17 mm^3^, which was significantly smaller than that of the saline group (1248.51 mm^3^, *P* < 0.0001). The tumor weight (Fig. 5J) and the tumor growth inhibition value (Fig. 5K) showed a similar trend to the tumor volume in each group. Representative photographs of the excised tumors at the study endpoint have been shown in the Supporting Information Fig. S8. In addition, within 20 days, the TP group showed the lowest survival rate, while there was no death in the NYP group (Fig. 5L), indicating that oral TP had serious toxic side effects, which were greatly alleviated by GA assembly and yeast capsule loading. It is worth noting that mice administered with YC and NYP showed a significantly increased number of Peyer's patches compared to the saline group (Fig. 5M), which was consistent with literature reports^33^. Since the gastrointestinal route for YC-mediated absorption and translocation involves M cells located in Peyer's patches, this increase is beneficial for enhanced transepithelial transport^44^.

To further confirm the antitumor effects at the histological level, we excised tumors and prepared H&E and immunofluorescence staining. As shown in Fig. 5N, tumor cells in the saline and GA groups were histiocytoid in shape and arranged tightly with complete structures. The TP containing formulation treatment groups observed apoptosis or necrosis of tumor cells, among which NYP showed the most representative apoptotic damage with disorganized tumor cell architecture and pyknotic nuclei. TUNEL staining revealed pronounced apoptotic cell death in tumor tissues, with the NYP group demonstrating the highest density of positive signals (Fig. 5O), which was consistent with the abovementioned animal experiment results. Increased MMP-9 expression promotes metastasis^45^. Therefore, we evaluated metastasis by measuring MMP-9 expression levels. As demonstrated in Fig. 5P, MMP-9 expression in tumor tissues was reduced across all treatment groups compared to the saline control. Notably, the GA group exhibited a slight decrease in MMP-9 levels relative to the saline and YC groups. More pronounced downregulation was observed in the NP groups, with NYP showing the most significant reduction among all treatments. VEGF promotes tumor angiogenesis^46^, and CD31 serves as a specific marker for vascular endothelial cells, enabling the assessment of tumor angiogenesis^47^. We also found that NYP effectively reduces the expression of CD31 and VEGF (Fig. 5Q and R). To investigate the molecular mechanism of TP-induced ICD, we also examined the cGAS–STING pathway, which plays a pivotal role in initiating innate immune responses following DNA damage. As presented in Supporting Information Fig. S9, TP treatment activated the cGAS–STING signaling axis, providing a mechanistic link between TP-induced ICD and subsequent antitumor immune activation. Collectively, the TME modulatory effect of NYP is attributed to the synergistic effect of ICD in TP with immunomodulatory effect of GA. Among all treatment groups, NYP demonstrated the strongest inhibitory effect on tumor invasion, metastasis, and angiogenesis.

### 3.5 NYP vaccines induce immune response and remodel the TME

To investigate oral vaccine-induced *in vivo* immune response, FCM was performed to analyze immune subsets in tumor tissues, including CD3^+^CD8^+^ T cells, CD3^+^CD4^+^ T cells, CD80^+^CD86^+^CD11c^+^ DCs, CD11b^+^Gr1^+^ MDSCs, CD3^+^CD4^+^CD25^+^ regulatory T cells (Tregs), and CD11b^+^ F4/80^+^ macrophages. Compared to the saline group, oral NYP vaccine significantly increased tumor infiltration of CD3^+^CD8^+^ T cells (6.62%) (Fig. 6A and B), CD3^+^CD4^+^ T cells (30.10%) (Fig. 6C and D), and CD80^+^CD86^+^CD11c^+^ DCs (39.20%) (Fig. 6E), with NYP showing a superior rate over GA, NP and YC groups. NYP also elevated the M1/M2 macrophage ratio (CD86^+^/CD206^+^) among CD11b^+^F4/80^+^ macrophages (1.33), which effectively induced the transition from M2-type to M1-type (Fig. 6F). And the proportion of Tregs in the NYP group was the lowest (10.16%, Fig. 6G). Suppression of these Tregs blocked the immunosuppressive effect of tumor cells. NYP-treated mice exhibited the lowest CD11b^+^Gr1^+^ MDSC infiltration (10.15%) (Fig. 6H). All these results indicated that NYP treatment dramatically reversed the immunosuppressive tumor microenvironment by upregulating immunostimulatory cells and downregulating the immunosuppressive cells.

**Figure 6:**
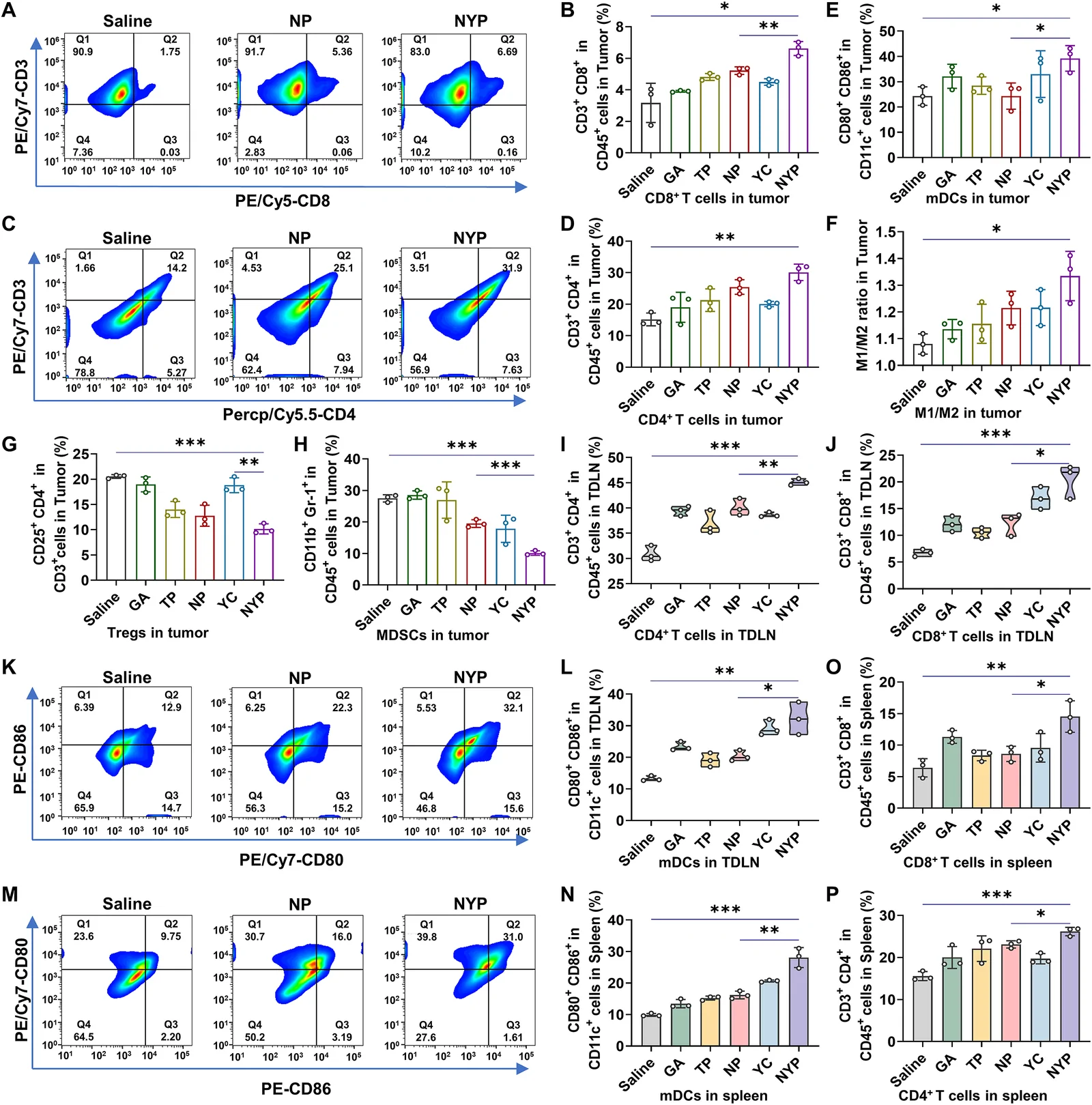
Immune response assessment of NYP *in vivo*. (A, B) Representative FCM plots and quantitative analysis of CD3^+^CD8^+^ T cells in the tumor (*n* = 3). (C, D) Representative FCM plots and quantitative analysis of CD3^+^CD4^+^ T cells in tumor (*n* = 3). (E) Quantitative analysis of CD80^+^CD86^+^CD11c^+^ DCs in tumor (*n* = 3). (F) M1/M2 macrophage ratio (CD86^+^/CD206^+^) within CD11b^+^F4/80^+^ macrophages in tumor (*n* = 3). (G) Quantitative analysis of CD3^+^CD4^+^CD25^+^ Tregs in tumor (*n* = 3). (H) Quantitative analysis of CD11b^+^Gr1^+^ MDSCs in tumor (*n* = 3). (I) Quantitative analysis of CD3^+^CD4^+^ T cells in TDLN. (J) Quantitative analysis of CD3^+^CD8^+^ T cells in TDLN. (K, L) Representative FCM plots and quantitative analysis of CD80^+^CD86^+^CD11c^+^ DCs in TDLNs. (M, N) Representative FCM plots and quantitative analysis of CD80^+^CD86^+^CD11c^+^ DCs in the spleen. (O) Quantitative analysis of CD3^+^CD8^+^ T cells in the spleen. (P) Quantitative analysis of CD3^+^CD4^+^ T cells in the spleen. Data are presented as mean ± SD. Statistical significance was determined by A one-way two-sided ANOVA. Specific *P* values are marked in the figure, with significance levels defined as ∗*P* < 0.05, ∗∗*P* < 0.01, ∗∗∗*P* < 0.001.

Further analysis of TDLNs revealed that, compared to the saline group (38.87% and 6.72%), GA increased the proportions of CD3^+^CD4^+^ T cells (39.43%) and CD3^+^CD8^+^ T cells (12.10%) in TDLNs, and the NYP group had significantly higher proportions of CD3^+^CD4^+^ (45.10%) and CD3^+^CD8^+^ T cells (20.43%) (Fig. 6I and J). And NYP elevated the proportion of mature DCs in TDLNs (Fig. 6K and L) with an average percentage of 32.27% CD80^+^CD86^+^ DCs compared to 13.27% in the saline group, and the proportional changes of mDCs, CD8^+^ T cells, and CD4^+^ T cells among splenic immune cells all exhibit the same trend (Fig. 6M–P), demonstrating NYP's efficacy in activating DCs and T cells in lymphoid organs.

It is noteworthy that while GA treatment enhanced the CD8^+^/CD4^+^ T cell ratio in both TDLNs and the spleen, and promoted DCs maturation in TDLNs, it demonstrated inefficient infiltration into tumor tissues. These findings indicate that although GA possesses intrinsic immunomodulatory capabilities, its impaired tumor-homing capacity consequently limits the antitumor efficacy.

In this strategy, TP was utilized as an ICD inducer to release tumor antigens and successfully improved the immunogenicity of tumor cells, which has been demonstrated above. Thus, the expression of ICD-related DAMPs (CRT and HMGB1) in tumor tissues was further analyzed *via* immunofluorescence staining. Consistent with the ICD effect observed *in vitro*, NYP-treated tumors exhibited the weakest red fluorescence in the nucleus, indicating that HMGB1 can be released extracellularly. And the strongest green fluorescence was observed in the NYP group, suggesting maximal CRT exposure (Fig. 7A), demonstrating NYP's superior capacity to induce ICD *in vivo*. As shown in Fig. 7B and C, the quantitative results of immunofluorescence staining were consistent with the observations from the representative images. This ICD-driven immunomodulation further amplified effector T cell activation, thereby potentiating anti-tumor immunity.

**Figure 7:**
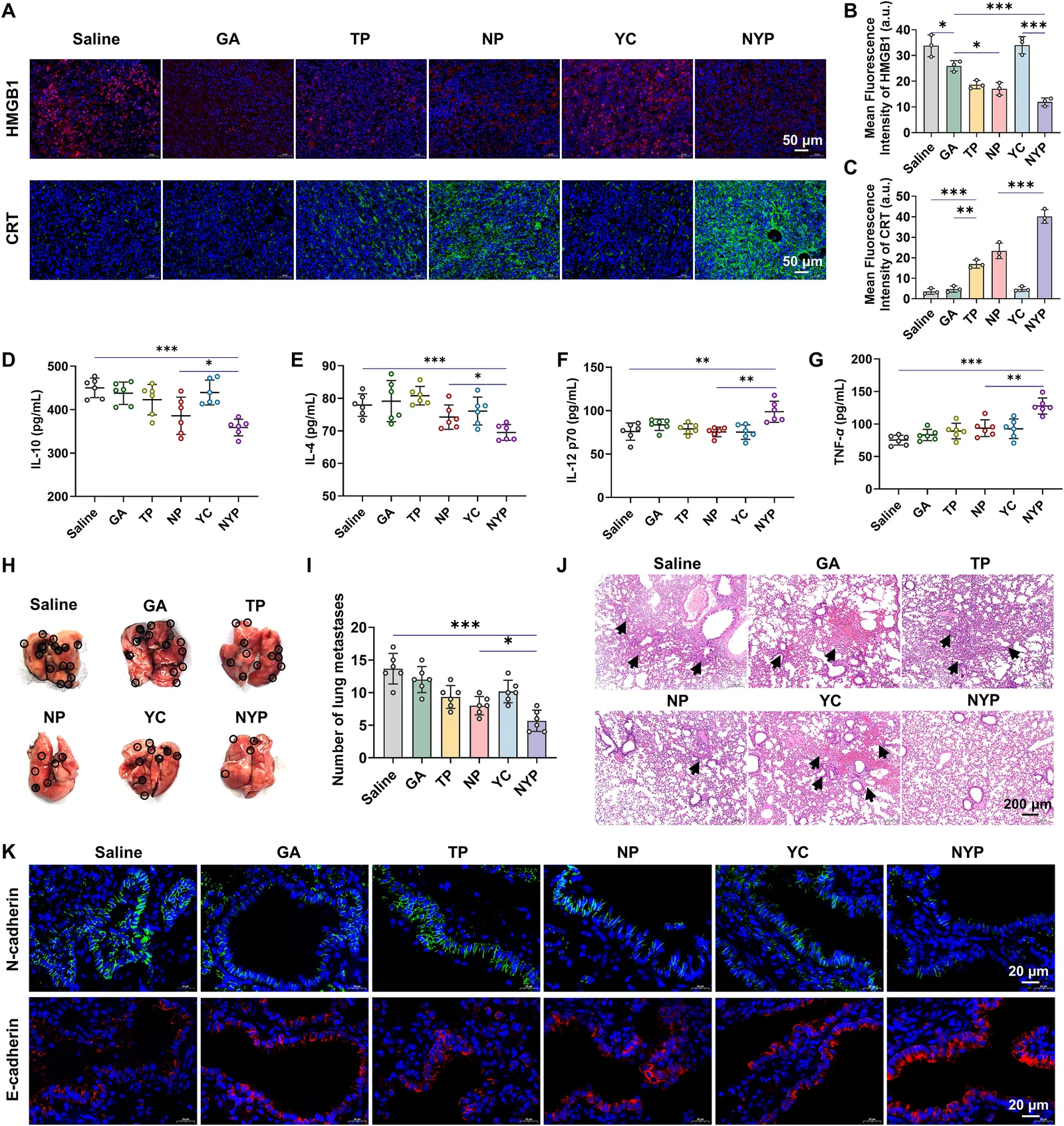
*In vivo* ICD activation, cytokine modulation and suppression of pulmonary metastasis. (A) Expression of HMBG1 (red) and CRT (green) in tumor tissue, cell nuclei were stained by DAPI (blue). (B, C) Quantification of mean fluorescence intensity of HMGB1 and CRT. Data were analyzed using ImageJ software (*n* = 3). (D, E) The levels of immunosuppressive cytokines (IL-10 and IL-4) in tumors was measured by ELISA kits (*n* = 6). (F, G) The levels of immunostimulatory cytokines (IL-12 p70 and TNF-*α*) in tumors was measured by ELISA (*n* = 6). (H) The lungs were photographed, and metastases are circled in black circles. (I) Statistics of the number of pulmonary metastatic nodules (*n* = 6). (J) Lung metastases were assessed by H&E staining of the lungs. (K) Expression of N-cadherin (green) and E-cadherin (red) in lung tissue, cell nuclei were stained by DAPI (blue). Data are presented as mean ± SD. Statistical significance was determined by A one-way two-sided ANOVA. Specific *P* values are marked in the figure, with significance levels defined as ∗*P* < 0.05, ∗∗*P* < 0.01, ∗∗∗*P* < 0.001.

Cytokines, acting as critical signaling released by immune cells in the TME, exhibit level alterations closely associated with the regulation of tumor immunity. To investigate the mechanism by which NYP reprograms the tumor immune microenvironment, we analyzed key immunomodulatory cytokines, including IL-10, IL-4, IL-12 p70, and TNF-*α*^48^^,^^49^. Supernatants from tumor homogenates were collected, and cytokine levels were quantified *via* ELISA assays. Compared to saline, NYP treatment significantly increased IL-12 p70 and TNF-*α* levels while suppressing IL-10 and IL-4 expression (Fig. 7D–G). It can be seen that NYP can activate the inflammatory response process for enhanced immunotherapy.

Next, we further evaluated whether the NYP vaccine could prevent tumor metastasis after remodeling the tumor immunosuppressive environment. Lung tissues were imaged, and we quantified metastatic nodules in the lung tissues. In contrast to the saline, GA, and YC groups exhibiting dense metastasis, NYP treatment significantly reduced pulmonary metastasis (Fig. 7H), and the pulmonary nodule count was significantly reduced (Fig. 7I), demonstrating a significant anti-metastatic effect. Consistent results were observed in H&E-stained lung sections (Fig. 7J), demonstrating superior protective efficacy against tumor dissemination. These results confirmed that the oral NYP vaccine induces potent immunological responses, effectively suppressing the pulmonary metastasis of tumor cells. We have performed immunofluorescence staining for two key EMT-related markers including N-cadherin (green) and E-cadherin (red), on lung tissue sections from different treatment groups. As shown in Fig. 7K, NYP treatment significantly increased E-cadherin expression while decreasing N-cadherin expression compared to the control group, indicating effective suppression of EMT process and thus suppressing lung metastasis.

### 3.6 NYP vaccines prevent tumor recurrence

In addition to activating systemic immunity to inhibit tumor metastasis, immunotherapy also establishes long-term immunological memory to suppress tumor recurrence^50^. Recurrence following surgical resection often compromises therapeutic outcomes clinically. Therefore, we combined surgical therapy to investigate the potential of these therapeutic interventions in preventing postoperative tumor recurrence. Following four-dose administration, 90% of the tumor mass was surgically resected (10% residual retained) (Fig. 8A). Subsequently, we assessed the capacity of these different therapeutic regimens to inhibit tumor recurrence. Compared to the saline, GA, TP, and YC groups, NYP-treated mice demonstrated a significantly reduced tumor growth rate with tumors eventually controlled, whereas uncontrolled tumor proliferation was observed in the saline group (Fig. 8B). Meanwhile, the results of tumor weight also verified this result (Fig. 8C). No significant differences in body weight were observed among groups during the treatment period, with minor fluctuations attributable to postsurgical weight loss following tumor resection (Supporting Information Fig. S10). In comparison to the saline and TP group, the group pre-surgically treated with NYP exhibited the highest survival rate with 90% within 24 days, indicating that tumor proliferation was better inhibited (Fig. 8D). All these results demonstrated the effectiveness of NYP oral vaccine combined with surgery.

**Figure 8:**
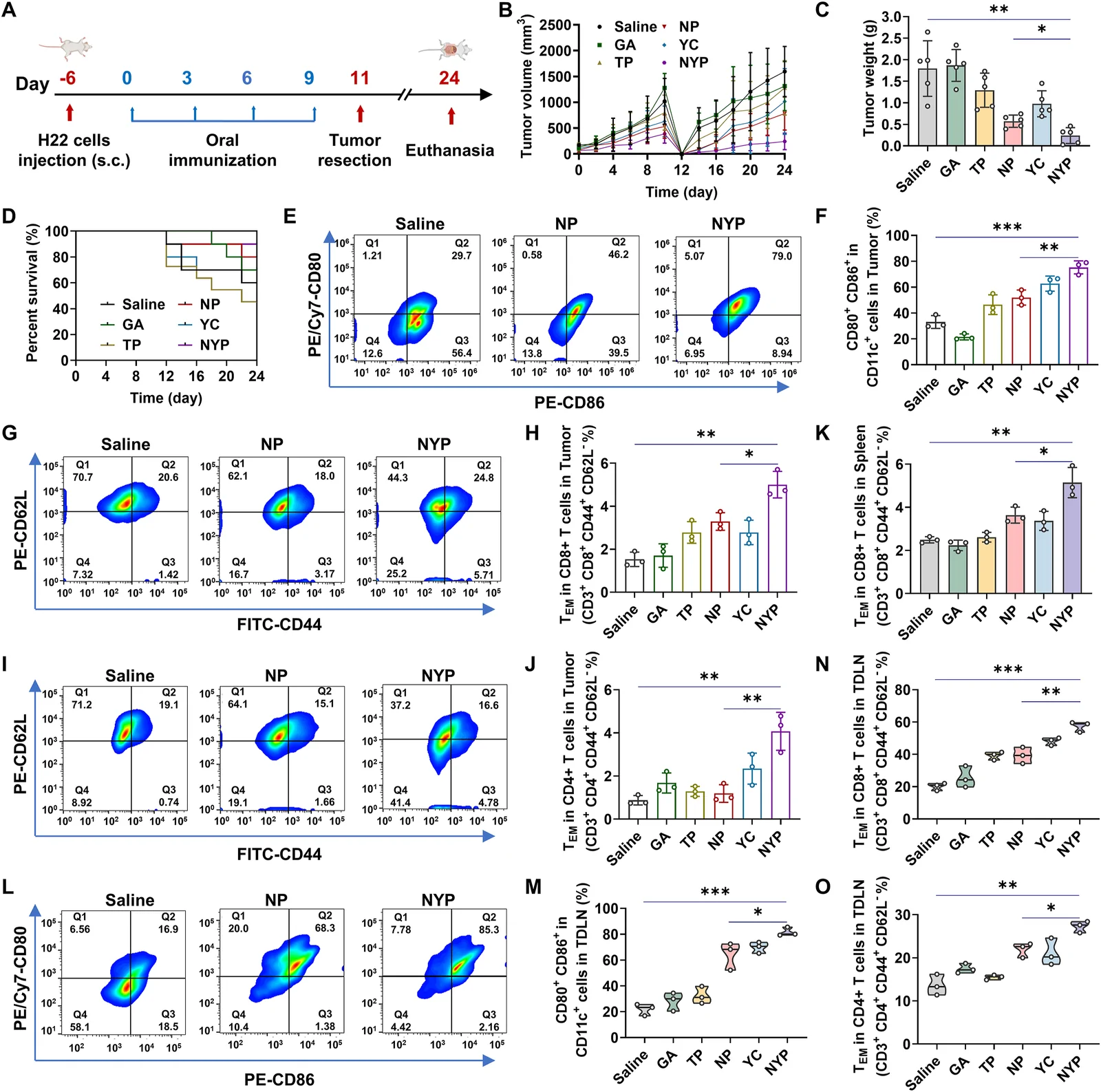
*In vivo* tumor recurrence prevention and immunological memory analysis. (A) Dosing regimens of recurrence prevention research. (B) Tumor growth curve (*n* = 5). (C) Tumor weight in each group (*n* = 5). (D) Survival rate of mice. (*n* = 10). (E, F) Representative FCM plots and quantitative FCM analysis of mature DCs in the tumor (*n* = 3). (G, H) Representative FCM plots and quantitative analysis of CD8^+^ T_EM_ cells in the tumor (*n* = 3). (I, J) Representative FCM plots and quantitative analysis of CD4^+^ T_EM_ cells in the tumor (*n* = 3). (K) Quantitative FCM analysis of mature DCs in spleen (*n* = 3). (L, M) Representative FCM plots and quantitative FCM analysis of mature DCs in TDLN (*n* = 3). (N) Quantitative FCM analysis of CD8^+^ T_EM_ in TNLN (*n* = 3). (O) Quantitative FCM analysis of CD4^+^ T_EM_ in TNLN (*n* = 3). Data are presented as mean ± SD. Statistical significance was determined by A one-way two-sided ANOVA. Specific *P* values are marked in the figure, with significance levels defined as ∗*P* < 0.05, ∗∗*P* < 0.01, ∗∗∗*P* < 0.001.

We also analyzed immune cells in tumors, TDLNs, and spleens to investigate the mechanism of NYP in preventing tumor recurrence *in vivo*. We performed CD11c^+^, CD80^+^, and CD86^+^ staining in tumor tissues to evaluate DCs maturation *via* FCM. Compared to the other groups, the NYP group showed the highest mature DCs percentage (75.26%), indicating enhanced DCs recruitment and maturation (Fig. 8E and F). The frequency of effector memory CD8^+^/CD4^+^ T cells (T_EM_, CD3^+^ CD8^+^/CD4^+^ CD62L^−^CD44^+^) was also analyzed in the tumor. The percentage of CD8^+^ T_EM_ in the NYP group was 4.02- and 1.82-fold higher than those in the saline and NP groups, respectively (Fig. 8G and H). Similarly, CD4^+^ T_EM_ percentages increased by 6.46- and 2.88-fold compared to saline and NP groups (Fig. 8I and J). T_EM_ in the spleen of mice across different treatment groups was further examined. In the spleen, CD8^+^ T_EM_ cells in the NYP group exhibited a proportion of 5.14%, representing a twofold increase compared to the saline group (Fig. 8K). Analogous trends were observed in TDLNs, where the NYP group exhibited significantly elevated proportions of mature DCs and CD4^+^/CD8^+^ T_EM_ cells relative to other groups (Fig. 8L–O). Thus, all the datas revealed that the NYP tumor vaccine indeed has the potential to induce a memory effect in mice for effectively inhibiting tumor recurrence.

### 3.7 Biosafety assessment and protective effects against TP-related side effects

To evaluate the biosafety profile of the NYP vaccine, healthy BALB/c mice were subjected to intragastric administration using different treatments. Each mouse received a total of four treatments (Fig. 9A). One day after the final administration, major organs, including hearts, livers, spleens, lungs, and kidneys, were harvested for H&E staining, while blood samples were collected for complete blood count and hepatic/renal function analysis. During the 12-day treatment, the body weight of mice was monitored, and no significant abnormalities were observed in all groups (Fig. 9B). Due to the high toxicity and strong irritation of TP, the potential gastrointestinal mucosal damage caused by oral administration cannot be ignored. Analysis of gastric H&E staining (Fig. 9C) showed discontinuous gastric mucosal surfaces with superficial depressions in the free TP group, reduced gastric fundic glands in both free TP and NP groups, while NYP exhibited morphology similar to the saline group with minimal damage. In the ileum and colon, the free TP group displayed disorganized ileum villi, reduced crypts, loosely arranged colonic epithelial cells, increased apoptotic bodies, partial mucosal loss, and severe crypt destruction. In contrast, the NYP group maintained clearer and more regularly arranged structures (Fig. 9D and E), indicating that after self-assembly with GA into nanoparticles and loading into the YC, it was transported to the tumor site through the Peyer's lymph nodes, resulting enhanced targeting effect of TP to tumor cells, thereby reducing gastrointestinal damage in NYP.

**Figure 9:**
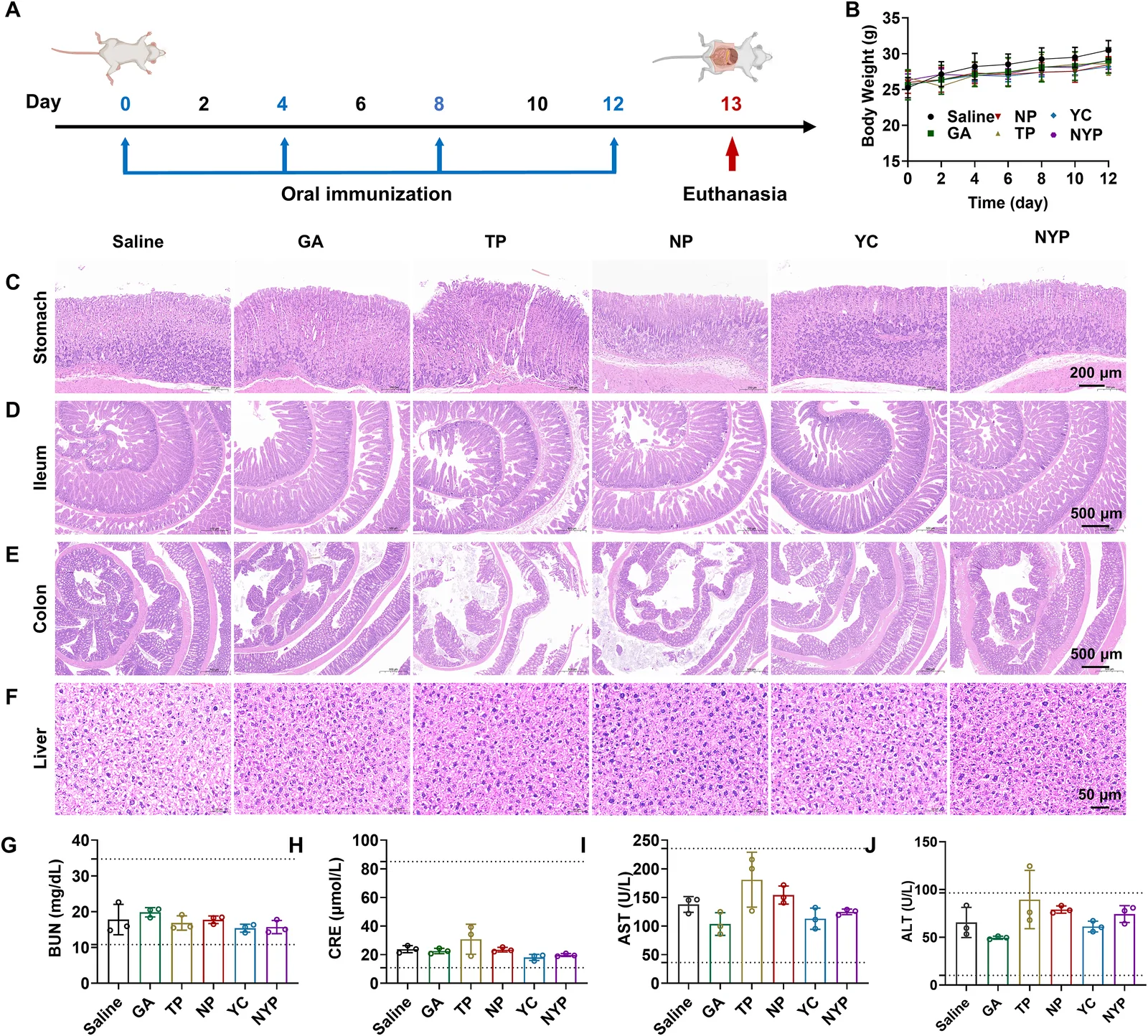
Biosafety assessment and protective effects against TP-related side effects. (A) Dosing regimens for safety evaluation. (B) Body weight changes in mice during 12-day administration. (C–F) H&E staining results of the stomach, ileum, colon and liver. (G–J) Serum biochemical parameters, including BUN, CRE, AST, and ALT (*n* = 3). Data are presented as mean ± SD. Statistical significance was determined by A one-way two-sided ANOVA. Specific *P* values are marked in the figure, with significance levels defined as ∗*P* < 0.05, ∗∗*P* < 0.01, ∗∗∗*P* < 0.001.

Additionally, H&E staining analysis of major organs was performed to evaluate the systemic toxicity of NYP. Liver H&E staining results demonstrated significant hepatic injury in the TP group, characterized by distinct karyorrhexis and karyolysis. Whereas the NYP group exhibited cellular morphology similar to the saline group, indicating that YC encapsulation effectively reduced the hepatotoxicity of TP (Fig. 9F). No obvious pathological damage was observed in other organs (including heart, spleen, lung, and kidney) upon histological examination (Supporting Information Fig. S11). The hematological parameters of mice, including WBCs, PLTs, Lymphocytes, Monocytes, HGB, Granulocytes, and RBCs, showed no significant hematological abnormalities (Supporting Information Fig. S12). Their biochemical profiles, such as BUN, CRE, AST, and ALT., were found to be comparable to those of the control group that received saline (Fig. 9G–J). The**s**e data demonstrate that, in healthy mice, NYP exhibits a favorable safety profile at the administered dose.

## 4 Discussion

Liver cancer remains one of the most lethal malignancies worldwide, with extremely limited curative options for patients with advanced disease. Current systemic therapies, dominated by targeted agents^51^ and immune checkpoint inhibitors^52^, are plagued by a core clinical bottleneck: an objective response rate below 30%, frequent acquired drug resistance, and severe systemic toxicities. These limitations are largely rooted in the inherent “immune-cold” phenotype, characterized by insufficient effector T cell infiltration and massive enrichment of immunosuppressive cells in the TME^53^. Meanwhile, conventional injectable tumor vaccines face critical challenges in breaking through the intestinal barrier and effectively activating mucosal immunity, failing to elicit robust and safe anti-tumor immune responses. To address these challenges, we developed an oral in situ tumor vaccine (TP/GA-NYPs) based on natural YC, establishing a full-chain therapeutic strategy of gastrointestinal stability–intestinal barrier breakthrough–lymphatic systemic transport–active tumor targeting–cascade immune activation. After oral administration, the rigid YC shell protects the micron-sized formulation from premature degradation and drug leakage in the harsh acidic and enzymatic gastrointestinal environment.

Upon reaching the ileal Peyer's patches, *β*-glucan on the YC surface is specifically recognized by the Dectin-1 receptor highly expressed on intestinal M cells, mediating efficient transepithelial transport across the intestinal barrier into the subepithelial region of Peyer's patches. Subsequently, NYP is efficiently phagocytosed by resident macrophages in the gut-associated lymphoid tissue; these NYP-laden macrophages act as “Trojan horses”, migrating through lymphatic vessels to the thoracic duct and then into the systemic circulation, and actively home to the liver tumor site *via* their inherent chemotaxis toward the immunosuppressive TME. After internalization by tumor cells, NYP is shuttled to the acidic lysosomal compartment, where the YC shell is degraded by *β*-glucosidase, triggering on-demand site-specific release of the loaded active agents. In this formulation, YC also acts as natural immune adjuvant to promote antigen-presenting cell maturation. TP efficiently induces tumor ICD, triggers in situ release of endogenous tumor neoantigens and damage-associated molecular patterns, and activates the cGAS–STING pathway to initiate tumor-specific anti-tumor immunity. GA downregulates metastasis-related MMP9, blocking tumor invasion and metastasis, and collaboratively reversing the immunosuppressive TME.

This oral vaccine ultimately achieves deep TME remodeling, with significantly increased cytotoxic CD8^+^ T cell infiltration and reduced accumulation of regulatory T cells and M2-type tumor-associated macrophages in tumor tissues. In immunocompetent mouse models, TP/GA-NYP exerted potent tumor growth inhibition, and simultaneously markedly suppressed lung metastasis and postoperative tumor recurrence. Notably, NYP exhibits promising synergistic potential in combination with anti-PD-1 immunotherapy, as its robust TME reprogramming and efficient tumor-specific T cell priming can effectively overcome the primary resistance to anti-PD-1 monotherapy in immune-cold liver cancer^54^, ^55^, ^56^, ^57^. Collectively, this study provides a novel design paradigm for oral liver cancer vaccines. Follow-up studies will complete systematic preclinical evaluation *via* orthotopic liver cancer models, to advance clinical translation and provide a promising new therapeutic solution for advanced liver cancer immunotherapy.

## Author contributions

Qian Cheng and Siyao Li contributed equally to this work. Qian Cheng: Conceptualization, Data curation, Methodology, Project administration, Funding acquisition, Writing – review & editing. Siyao Li: Methodology, Validation, Data curation, Visualization, Writing – original draft. Lu Deng: Visualization, Investigation. Li Xiong: Methodology. Huiling Hu: Resources. Jingjing Li: Software. Lele Zhang: Formal analysis. Boda Guo: Methodology. Chuan Zheng: Supervision. Jinming Zhang: Methodology, Resources, Funding acquisition, Supervision.

## Conflicts of interest

The authors declare no conflicts of interest.

## Appendix A Supporting information

The following is the Supporting Information to this article:
