# Supplementary material for: Oral artificial microbio-vaccine triggers synergistic immunogenic cell death induction and tumor microenvironment remodeling for liver cancer therapy
Source: Acta Pharm Sin B. 2026 Jul 4;16(10):6924–42. doi: 10.1016/j.apsb.2026.06.058 (PMC13712360; doi:10.1016/j.apsb.2026.06.058)
Supplement: Supplementary file 1 [file mmc1.docx]

**Supporting Information for**

**Original article**

**Oral artificial microbio-vaccine triggers synergistic immunogenic cell death induction and tumor microenvironment remodeling for liver cancer therapy**

**Qian Cheng^a,†^, Siyao Li^a,†^, Lu Deng^a^, Li Xiong^a^, Huiling Hu^a^,** **Jingjing Li^e^, Lele Zhang^d,*^, Boda Guo^c,*^, Chuan Zheng^b,*^, Jinming Zhang****^a,*^**

^a^*Chinese Medicine Germplasm Resources Innovation and Effective Uses Key Laboratory of Sichuan Province, Chengdu University of Traditional Chinese Medicine, Chengdu 611130, China*

^b^*TCM Regulating Metabolic Diseases Key Laboratory of Sichuan Province, Hospital of Chengdu University of Traditional Chinese Medicine, Chengdu 610072, China*

^c^*Department of Urology, Peking University Third Hospital, Beijing 100191, China*

^d^*School of Preclinical Medicine, Chengdu University, Chengdu 610106, China*

^e^*Department of Rehabilitation Sciences, Facuslty of Health and Social Sciences, Hong Kong Polytechnic University, Hong Kong 999077, China*

Received 24 January 2026; received in revised form 24 March 2026; accepted 12 April 2026

^†^These authors contributed equally to this work.

*Corresponding authors.

E-mail: [cdutcmzjm@126.com](mailto:cdutcmzjm@126.com) (Jinming Zhang); [zhengchuan@cdutcm.edu.cn](mailto:zhengchuan@cdutcm.edu.cn) (Chuan Zheng); [guoboda@126.com](mailto:guoboda@126.com) (Boda Guo); [zhanglele@cdu.edu.cn](mailto:zhanglele@cdu.edu.cn) (Lele Zhang).

1. **Supporting figures**

**Figure S1** *In vitro* release profiles of TP from NP and NYP in SIF (pH 6.8) and SGF (pH 1.2) at 37 ℃ (*n* = 3).

**Figure S2** *In vitro* release profiles of TP and GA from NP and NYP in pH 1.2, pH 4.5, and pH 7.4 solutions at 37 ℃ (*n* = 3).

**Figure S3** Uptake of NYP by RAW264.7 cells (*n*=3). Representative confocal images (left) and flow cytometric quantification (right) of NYP internalization after 4 h incubation.

**Figure S4** Uptake of NYP by HepG2 cells (*n*= 3). Representative confocal images (left) and flow cytometric quantification (right) of NYP internalization after 8 h incubation.

**Figure S5** Cell viability of RAW264.7 and Caco-2 cells presented as percentage of control after treatment with TP and NYP after 24 h (*n* = 5).

**Figure S6** *In vitro* ICD effect in HepG2. (A, B) Representative FCM histogram and quantitative analysis of HMGB1 release (*n* = 3). (C, D) Representative FCM histogram and quantitative analysis of CRT exposure on the cell surface (*n* = 3). (E) Intracellular ATP concentrations of HepG2 cells after incubation with different formulations for 24 h, measured by a luminescence-based assay (*n* = 3). Data are presented as mean ± SD. Statistical significance was determined by A one-way two-sided ANOVA. Specific *P* values are marked in the figure, with significance levels defined as **P* < 0.05, ***P* < 0.01, ****P* < 0.001.

**Figure S7** Comparison of ICD-inducing effect between NYP and DOX in H22 cells. (A, B) Representative FCM histogram and quantitative analysis of HMGB1 release (*n* = 3). (C–E) Representative FCM plots, histogram and quantitative analysis of CRT exposure on the cell surface (*n* = 3). (F) Intracellular ATP levels in H22 cells measured by a luminescence-based assay (*n* = 3). Data are presented as mean ± SD. Statistical significance was determined by A one-way two-sided ANOVA. Specific *P* values are marked in the figure, with significance levels defined as **P* < 0.05, ***P* < 0.01, ****P* < 0.001, and ns denotes no significant difference.

**Figure S8** Representative photographs of tumors excised from each treatment group.

**Figure S9** Immunofluorescence analysis of cGAS and STING expression in tumor tissues from different treatment groups. Representative images showing cGAS (green) and STING (red) immunofluorescence staining in tumor sections harvested from mice in the indicated groups. Cell nuclei were stained with DAPI (blue).

**Figure S10** *In vivo* tumor recurrence prevention and immunological memory analysis. Weight change during the 24 days treatment in tumor recurrence prevention experiment (*n* = 5).

**Figure S11** Biosafety assessment and protective effects against TP-related side effects. H&E staining results of heart, spleen, lung, and kidney.

**Figure S12** Biosafety assessment and protective effects against TP-related side effects. Results of complete blood count (CBC) analysis (*n*=5); including white blood cell count (WBC), platelet count (PLT), lymphocyte, monocytes, hemoglobin (Hb), granulocyte, and red blood cell count (RBC).

2. **Surpporting tables**

**Table S1** EE and LE of TP and GA in NYP.

| Formulation | EE of TP (%) | LE of TP (%) | EE of GA (%) | LE of GA (%) |
| --- | --- | --- | --- | --- |
| TP/GA-NYPs | 65.26 ± 4.60 | 1.78 ± 7.60 | 58.97 ± 5.18 | 2.71 ± 7.47 |

Data are mean ± SD (*n*=3).
